# Molecular impact of nicotine and smoking exposure on the developing and adult mouse brain

**DOI:** 10.1101/2024.11.05.622149

**Published:** 2024-11-05

**Authors:** Daianna Gonzalez-Padilla, Nicholas J. Eagles, Marisol Cano, Geo Pertea, Andrew E. Jaffe, Kristen R. Maynard, Dana B. Hancock, James T. Handa, Keri Martinowich, Leonardo Collado-Torres

**Affiliations:** 1 Lieber Institute for Brain Development, Johns Hopkins Medical Campus, Baltimore, MD, 21205, USA; 2 Wilmer Eye Institute, Johns Hopkins School of Medicine, Baltimore, MD, 21287, USA; 3 The Solomon H. Snyder Department of Neuroscience, Johns Hopkins School of Medicine, Baltimore, MD, 21205, USA; 4 Department of Psychiatry and Behavioral Sciences, Johns Hopkins School of Medicine, Baltimore, MD, 21205, USA; 5 RTI International, Research Triangle Park, NC, 27709, USA; 6 Johns Hopkins Kavli Neuroscience Discovery Institute, Johns Hopkins University, Baltimore, MD, 21218, USA; 7 Department of Biostatistics, Johns Hopkins Bloomberg School of Public Health, Baltimore, MD, 21205, USA

## Abstract

Maternal smoking during pregnancy (MSDP) is associated with significant cognitive and behavioral effects on offspring. While neurodevelopmental outcomes have been studied for prenatal exposure to nicotine, the main psychoactive component of cigarette smoke, its contribution to MSDP effects has never been explored. Comparing the effects of these substances on molecular signaling in the prenatal and adult brain may provide insights into nicotinic and broader tobacco consequences that are developmental-stage specific or age-independent. Pregnant mice were administered nicotine or exposed to chronic cigarette smoke, and RNA-sequencing was performed on frontal cortices of postnatal day 0 pups born to these mice, as well as on frontal cortices and blood of the adult dams. We identified 1,010 and 4,165 differentially expressed genes (DEGs) in nicotine and smoking-exposed pup brains, respectively (FDR<0.05, Ns = 19 nicotine-exposed vs 23 vehicle-exposed; 46 smoking-exposed vs 49 controls). Prenatal nicotine exposure (PNE) alone was related to dopaminergic synapses and long-term synaptic depression, whereas MSDP was associated with the SNARE complex and vesicle transport. Both substances affected SMN-Sm protein complexes and postsynaptic endosomes. Analyses at the transcript, exon, and exon-exon junction levels supported gene level results and revealed additional smoking-affected processes. No DEGs at FDR<0.05 were found in adult mouse brain for any substance (12 nicotine-administered vs 11 vehicle-administered; 12 smoking-exposed vs 12 controls), nor in adult blood (12 smoking-exposed vs 12 controls), and only 3% and 6.41% of the DEGs in smoking-exposed pup brain replicated in smoking-exposed blood and human prenatal brain, respectively. Together, these results demonstrate variable but overlapping molecular effects of PNE and MSDP on the developing brain, and attenuated effects of both smoking and nicotine on adult versus fetal brain.

## INTRODUCTION

As of 2021, 4.6% of mothers in the United States smoked cigarettes during pregnancy. Although declining in prevalence over time, maternal smoking during pregnancy (MSDP) remains a major public health problem due to the risk it imposes on the health of hundreds of thousands of mothers and their offspring (1,2). Adverse health implications for pregnant women include increased risk for preterm deliveries and miscarriages, and impacts on lung and brain development from various toxic compounds in tobacco smoke for the unborn (3). Prenatal tobacco exposure is also associated with cognitive and behavioral disruption. Specifically, exposed babies are predisposed to impaired language and learning skills, attention deficits, conduct and behavioral alterations, and are at higher risk of developing substance use disorders (4). Several studies have investigated MSDP and prenatal nicotine exposure (PNE) in animal models and confirmed similar effects (4).

Because cigarette smoke contains a mixture of over 7,000 compounds (5), understanding the molecular mechanisms and cellular processes by which tobacco smoke affects neurodevelopment is complex. Many of these constituents are toxic or carcinogenic, and can disrupt brain function (6–10). However, little information is available regarding how individual components of cigarette smoke affect the developing brain during prenatal exposure. The most comprehensively studied substance is nicotine, the main psychoactive component of cigarette smoke. Nicotine activates and desensitizes nicotinic acetylcholine receptors (AChRs) in the developing central nervous system (CNS), and impacts brain development (4,11). Despite extensive data demonstrating a causal association between PNE and brain function (11), the extent to which PNE accounts for the effects of MSDP is not known. However, identifying the molecules and pathways driven by nicotine versus other components present in tobacco smoke is critical to understand impacts of MSDP on neurodevelopment. A transcriptomic investigation of the human prefrontal cortex from postmortem brain donors identified 14 MSDP-associated differentially expressed genes, but did not specifically assess effects of nicotine versus other substances (12). Model organisms can be useful to further study MSDP in controlled settings to untangle nicotine-specific contributions.

Here, we investigated molecular impacts of prenatal exposure in mice of both chronic cigarette smoke and nicotine on offspring (P0: postnatal day 0) as well as to the adult, exposed females compared to controls. Differential expression analysis of frontal cortex tissue revealed changes at the gene level when comparing exposed and unexposed pup brain samples. Affected features by prenatal nicotine and smoking exposure were contrasted and were compared against changes observed in adult brain. These results overlap with previous reports in human, identifying several convergent gene targets. Together, the findings suggest differential, but overlapping transcriptomic modifications from gestational exposure to nicotine and cigarette smoke on the developing brain. Novel PNE and MSDP-associated changes were identified in expression features beyond gene expression modifications, and variability in differential gene expression due to tobacco exposure (nicotine and cigarette smoke) across age (prenatal or adult brain), tissue (brain or blood), and species (human or mouse brain) was noted.

## MATERIALS AND METHODS

Detailed materials and methods can be found in Supplementary Materials and Methods.

### Samples

**Figure 1:**
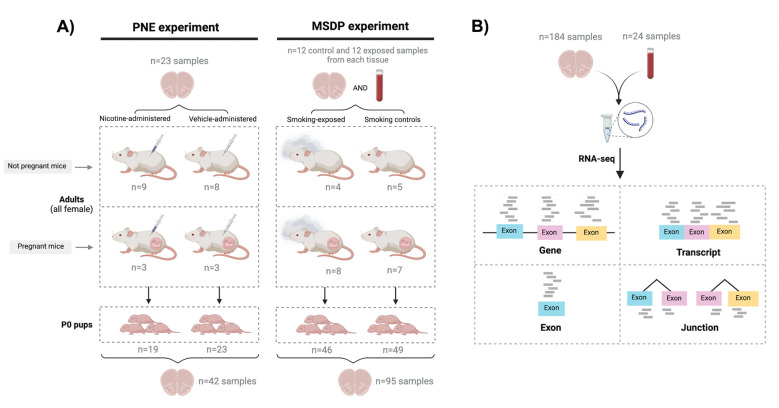
Experimental design of the study. **A)** 21 pregnant mice were split into two experiments: in the first one prenatal nicotine exposure (PNE) was modeled administering nicotine (n=3) or vehicle (n=3) to the dams during gestation, and in the second maternal smoking during pregnancy (MSDP) was modeled exposing dams to cigarette smoke during gestation (n=8) or using them as controls (n=7). A total of 137 pups were born: 19 were born to nicotine-administered mice, 23 to vehicle-administered mice, 46 to smoking-exposed mice, and 49 to smoking control mice. 17 nonpregnant adult females were also nicotine-administered (n=9) or vehicle-administered (n=8) to model adult nicotine exposure, and 9 additional nonpregnant dams were smoking-exposed (n=4) or controls (n=5) to model adult smoking. Frontal cortex samples of all P0 pups (n=137: 42 for PNE and 95 for MSDP) and adults (n=47: 23 for the nicotine experiment and 24 for the smoking experiment) were obtained, as well as blood samples from the smoking-exposed and smoking control adults (n=24), totaling 208 samples. Number of donors and samples are indicated in the figure. **B)** RNA was extracted from such samples and bulk RNA-seq experiments were performed, obtaining expression counts for genes, transcripts, exons, and exon-exon junctions.

The frontal cortex was isolated from P0 offspring and adult females that delivered the pups, across two separate experiments: one for gestational smoking, and one for nicotine administered during gestation. Blood samples were collected from all smoking-exposed and control adults. In total, 208 samples were collected: 184 brain samples and 24 blood samples (Fig. 1A, Table S1). We isolated total RNA and performed bulk RNA sequencing (Fig. 1B; Supplementary Materials and Methods).

### RNA-seq data processing and exploration

Raw sequencing reads were pre-processed and aligned with *SPEAQeasy* (13) and used for expression quantification of genes, transcripts, exons, and exon-exon junctions (Fig. 1B). After normalizing read counts and filtering out lowly-expressed features (Fig. S1), samples were separated by tissue and age (Fig. S2, Fig. S3) and filtered by quality control metrics (Fig. S4). Dimensionality reduction analysis identified poor-quality samples that were further removed (Fig. S5, Fig. S6), and revealed transcriptomic sample differences driven by experiment among adult brains (Fig. S5, Fig. S7A, Fig. S8), by sex among pup brains (Fig. S6, Fig. S7B, Fig. S9), and by pregnancy in blood (Fig. S3B, Fig. S10). After discarding poor-quality samples, 23 blood samples, 39 adult brain samples, and 130 pup brain samples were used (Table S2).

Additional sample-level sources of gene expression variation were identified through variance partition and canonical correlation analyses, which informed the design of the statistical models used for differential expression analysis (DEA) (Fig. S11, Fig. S12).

### Differential Expression Analysis (DEA)

Five differential gene expression analyses were performed under the empirical Bayesian framework of *limma*-*voom* (14), comparing 1) nicotine vs vehicle exposure in pup brain, 2) smoking exposure vs control in pup brain, 2) nicotine vs vehicle administration in adult brain, 4) smoking exposure vs control in adult brain, and 5) smoking exposure vs control in adult blood (Fig. S13). Gene expression was adjusted for quality control metrics and batch effects, and by sex in pup brain, and pregnancy in adult brain and blood. DEA of expression features other than genes were performed for smoking and nicotine exposures in pup brain (Fig. S1). Only genes, transcripts, and exon-exon junctions with *p*-values adjusted for a false discovery rate (FDR) <5%, as well as exons with an FDR<5% and |log2FC|>0.25, were considered differentially expressed (DE).

Resulting moderated gene *t*-statistics were compared between experiments, ages, tissues, and against results from a previous transcriptomic study of prenatal and adult smoking exposure in human dorsolateral prefrontal cortex (12) (Fig. S1).

### Functional enrichment analysis

Genes annotated in Gene Ontology (GO) terms and in pathways of the Kyoto Encyclopedia of Genes and Genomes (KEGG), were assessed for their enrichment among our sets of genes applying one-sided Fisher’s exact tests, as implemented in *clusterProfiler* (15), and were FDR controlled.

## RESULTS

The frontal cortex was isolated from P0 offspring across two separate experiments: 1) pups born to female mice exposed to gestational smoking (n=46) or pups born to control female mice (n=49); 2) pups born to female mice administered nicotine during gestation (n=19) or pups born to female mice administered vehicle during gestation (n=23). The frontal cortex was also collected from the adult females that delivered the pups plus additional nonpregnant dams that were: 1) exposed to cigarette smoke (n=12; 8 pregnant) or smoking controls (n=12; 7 pregnant), and 2) administered nicotine (n=12; 3 pregnant) or vehicle-administered (n=11; 3 pregnant). Additionally, blood samples were collected from all smoking-exposed and control adults (n=24, Fig. 1A, Table S1). We isolated total RNA from all 208 samples and performed bulk RNA sequencing. From these data, we measured the transcriptome at four expression feature levels: genes, transcripts, exons, and exon-exon junctions (Fig. 1B). Poor-quality samples were discarded, resulting in a final study size of 130 pup brain samples, 39 adult brain samples, and 23 blood samples (n=192, Materials and Methods, Table S2, Fig. S1).

### Molecular impact of gestational exposure to nicotine and smoking on developing frontal cortex of offspring

From the frontal cortex of P0 offspring from both the prenatal nicotine exposure (PNE) and maternal smoking during pregnancy (MSDP) experiments, we performed differential expression analysis (DEA) at gene, transcript, exon, and exon-exon junction levels (Fig. 1, Fig. S1). Expression features other than genes were analyzed to support and complement gene-level inferences (16–18). Biologically, gene-level expression is composed by adding transcript-level expression, although gene-level RNA-seq quantification is performed by different computational methods (16,19,20). The highest expressed transcript in a gene can dominate gene-level expression measurements, masking out transcript-level changes (16–18). In addition, transcripts of the same gene with opposing expression directionalities can cancel each other out (16–18). Moreover, exons and exon-exon junction counts can provide additional insights into transcript abundances and alternative splicing (21–24).

**Figure 2:**
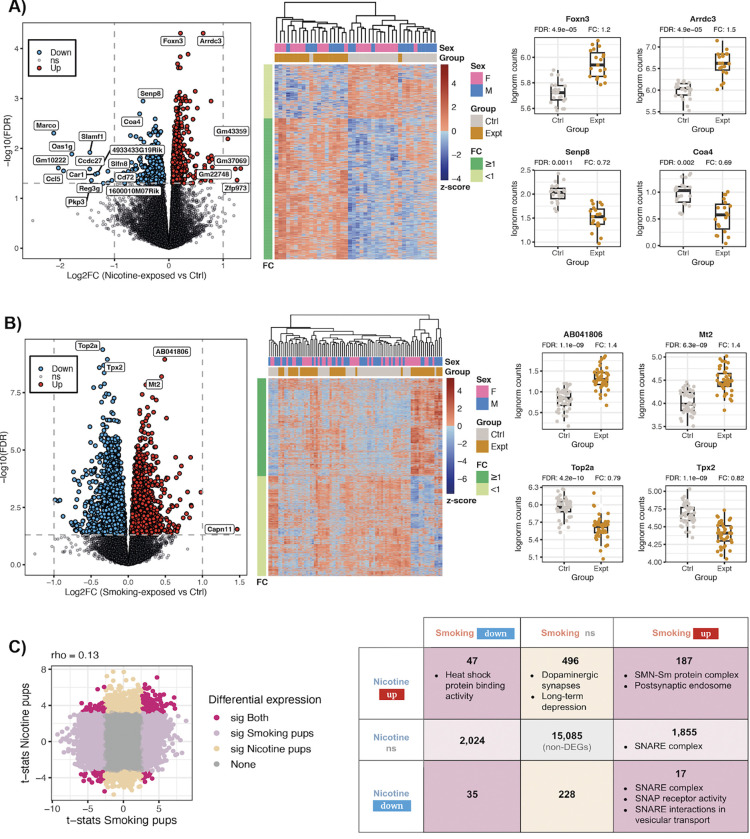
Differentially expressed genes in pup brain. Results of the differential gene expression analysis for **A)** prenatal nicotine vs vehicle exposure (PNE experiment) and **B)** prenatal smoking exposure vs control (MSDP experiment): volcano plots (left) show for each gene its log2-fold-change (logFC) and the −log10 of its false discovery rate (FDR) adjusted *p*-value for differential expression; in blue the DEGs (FDR<0.05) that were downregulated and in red the ones that were upregulated; non-significant (ns) genes appear in gray; labeled genes had |logFC|>1 or were the top 2 most significantly up- or down-regulated genes. Heat maps (middle) show the z-scores for the log2-CPM of the DEGs across samples; left color bars show the FC direction of the genes and top color bars the corresponding sex and experimental group of the samples. Box plots (right) show the log2-CPM of the top 2 most significant up- and down-regulated DEGs in control (Ctrl) and exposed samples (Expt). **C)** Scatter plot of the moderated *t*-statistics for differential expression of the genes for smoking and nicotine exposure. In dark pink the genes that were significantly DE under both exposures, in light pink and beige the ones that were significant for smoking or nicotine exposure only, respectively, and in gray genes that were not significant in any of the experiments; rho corresponds to the Spearman correlation coefficient. The right table presents the number of up- and down-regulated DEGs, as well as non-significant genes, for both nicotine and smoking exposures in pup brain. The molecular functions, cellular components and pathways that are significantly enriched in the given sets of DEGs are indicated. Related to Fig. S14, Fig. S15, Table S3, Table S4 and Table S5.

Comparing nicotine to vehicle exposure (PNE experiment), we identified 1,010 differentially expressed genes (DEGs, FDR<0.05) (Fig. 2A, Table S3); 280 DEGs were downregulated and 730 were upregulated. The top two most significantly up- and down-regulated genes were *Foxn3* and *Arrdc3,* and *Senp8* and *Coa4*, respectively (Fig. 2A). Comparing smoking exposure to control (MSDP experiment), 4,165 genes were differentially expressed (FDR<0.05): 2,106 were downregulated and 2,059 upregulated (Fig. 2B, Table S4). *Top2a* and *Tpx2* were the most significant DEGs and were downregulated, followed by the upregulated *AB041806* and *Mt2* genes (Fig. 2B). While differential gene expression (DGE) results were poorly correlated between the two experiments (rho=0.13, Fig. 2C), we identified 187 shared upregulated genes and 35 shared downregulated genes (Fig. 2C). Among the shared upregulated DEGs, *Strap, Snrpd3*, and *Snrpb* act in SMN-Sm protein complexes and *Nsg1*, *Clstn1*, and *Rab4a* in postsynaptic endosomes (Fig. S14A, Fig. S15A,B). Additionally, 496 genes were upregulated after nicotine exposure, but were not affected by cigarette smoke (Fig. 2C), of which 15 were associated with dopaminergic synapses and 8 with long-term synaptic depression (Fig. S14B, Fig. S15C,D). Similarly, 1,855 genes were upregulated after smoking exposure, but unaltered by nicotine (Fig. 2C), with 15 genes involved in the SNARE complex (Fig. S14A, Fig. S15E), which mediates neurotransmitter release. Furthermore, 17 DEGs were upregulated by smoking exposure and downregulated by nicotine exposure (Fig. 2C); of these, *Stx17* and *Bnip1* were enriched for the SNARE complex (Fig. S14A–C, Fig. S15F). 47 genes were upregulated by nicotine and downregulated by smoking (Fig. 2C), 4 showing enrichment for heat shock protein binding activity (Fig. S14C, Fig. S15G). A summary of the DGE results is provided in Table S5.

**Figure 3:**
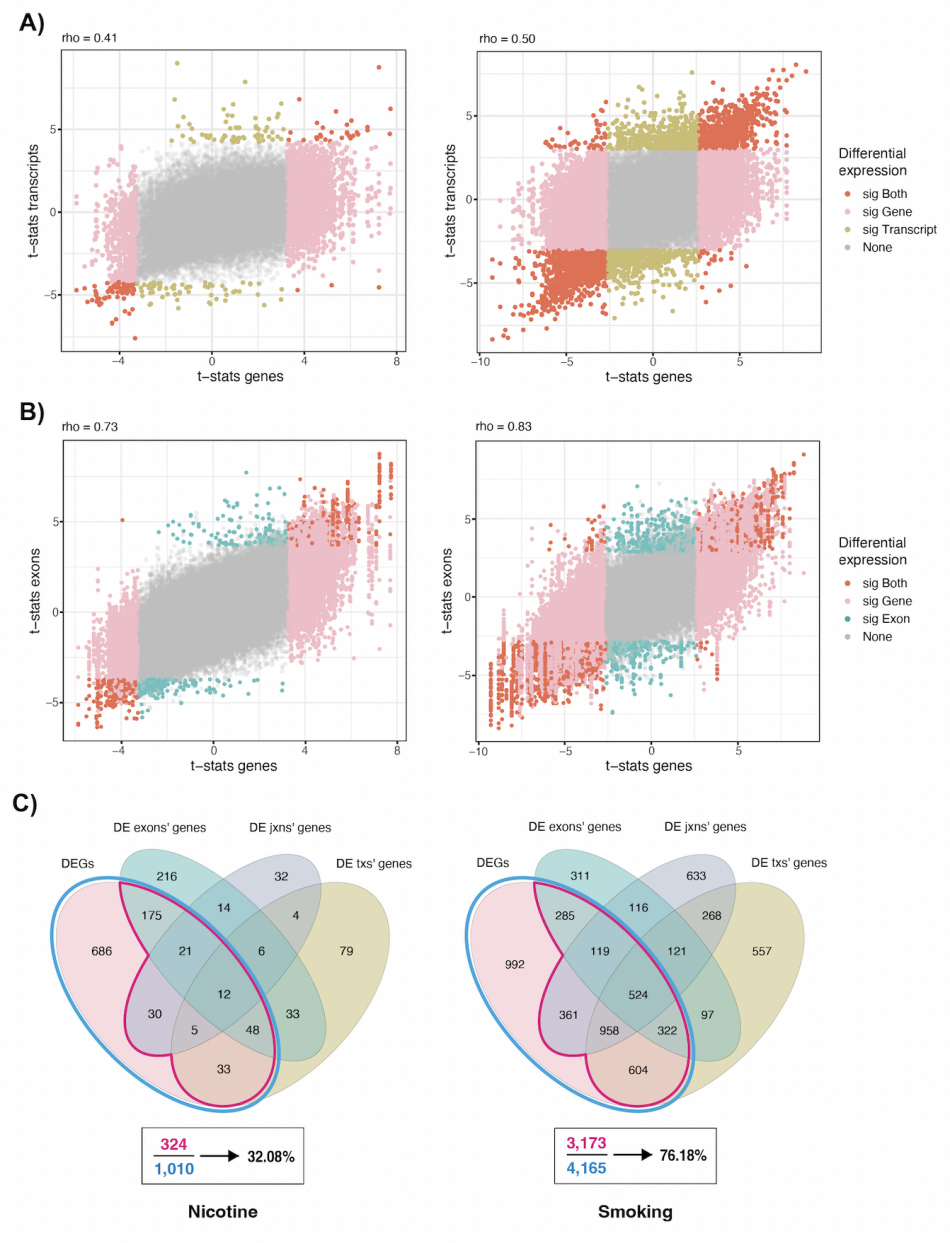
DEA results at gene, transcript, exon, and exon-exon junction levels in pup brain. Moderated *t*-statistics for differential expression of **A)** transcripts and **B)** exons in the nicotine (left) and smoking (right) experiments vs the moderated gene-level *t*-statistics in the same experiments. In dark orange DE features whose genes were also DE; in yellow and blue DE features of non-DEGs; in pink non-DE features of DEGs, and in gray non-DE features of non-DEGs. Rho corresponds to the Spearman correlation coefficient. DE transcripts and genes were defined with an FDR<5% and DE exons with FDR<5% and |logFC|>0.25. **C)** Overlap between DEGs and genes of DE transcripts (txs), exons, and exon-exon junctions (jxns) in the nicotine and smoking experiments. The percentages of DEGs with any other DE features are indicated. Related to Fig. S17, Table S8, Table S9, Table S12, Table S13 and Supplementary File 1.

Differential transcript expression (DTE) analysis identified 232 DE transcripts (FDR<0.05, mapping to 220 unique genes) for nicotine versus vehicle exposure (Table S6) and 4,059 DE transcripts (mapping to 3,451 unique genes) for smoking exposure versus control (Table S7, Fig. S16). Comparing DTE against DGE results for nicotine exposure, DE statistics were concordant at the gene and transcript levels (rho=0.41, Fig. 3A, Table S8), and most transcripts of DEGs were not differentially expressed, reflecting the transcript diversity for each gene. Similarly, for smoking exposure gene- and transcript-level DE statistics were concordant (rho=0.50, Fig. 3A, Table S9). However, some genes such as *Phf3, Ankrd11*, *Trpc4*, *Bcl11a*, *Scaf11, Dgcr8*, *Pnsir,* and *Dcun1d5* for nicotine exposure, and *Btf3*, *Cyhr1*, *H13*, *Srsf6, Meaf6*, *Ivns1abp*, *Morf4l2*, *Sin3b*, and *Ppp2r5c* for smoking exposure presented dissimilar DTE and DGE results (Fig. S17). Contrasting DTE results across exposures (Fig. S16), functional gene profiles for the DE transcripts corroborated and expanded DGE results (Fig. S14). We found 1,427 genes expressing upregulated transcripts under smoking exposure only, including 14 and 55 genes encoding for proteins associated with the SNARE complex and transport vesicles, respectively (Fig. S14E; Fig. S15E,H), as well as 68 genes involved in Parkinson’s, Huntington’s, or prion-related diseases (Fig. S14F, Fig. S15I).

Differential exon expression (DEE) analysis identified 1,115 DE exons (FDR<0.05 and |logFC|>0.25) for nicotine exposure (Table S10) and 5,983 DE exons for smoking exposure (Table S11). Similarly to DTE, there was a strong correlation between DEE and DGE statistics (rho=0.73 and 0.83 for nicotine and smoking exposure, respectively; Fig. 3B, Table S12, Table S13). DE analysis at the exon-exon junction level (DJE) revealed 205 DE junctions (FDR<0.05) for nicotine exposure (Table S14) and 9,515 DE junctions for smoking exposure (Table S15). Overall, we found agreement between DE analysis results at all four expression levels, with 32% and 76.18% of the DEGs for nicotine and smoking exposure, respectively, DE at least in one other feature level (Fig. 3C). Functional enrichment analysis for different sets of genes based on their DE signal at the different expression levels identified synaptic vesicle and membrane components as associated with the smoking exposure (Fig. S18A), and overall complemented the gene-only results (Fig. S14). DTE, DEE, and DJE results can be used to classify DEGs based on their support at these other expression feature levels (Fig. S19). More fine-grained agreement for the different exposures can also be assessed to select DEGs with additional support or focus on results missed by the DGE analysis (Fig. S20). Further DTE, DEE, and DJE results were identified (Supplementary File 1).

### Molecular impact of nicotine administration and smoking exposure on adult frontal cortex and blood

**Figure 4:**
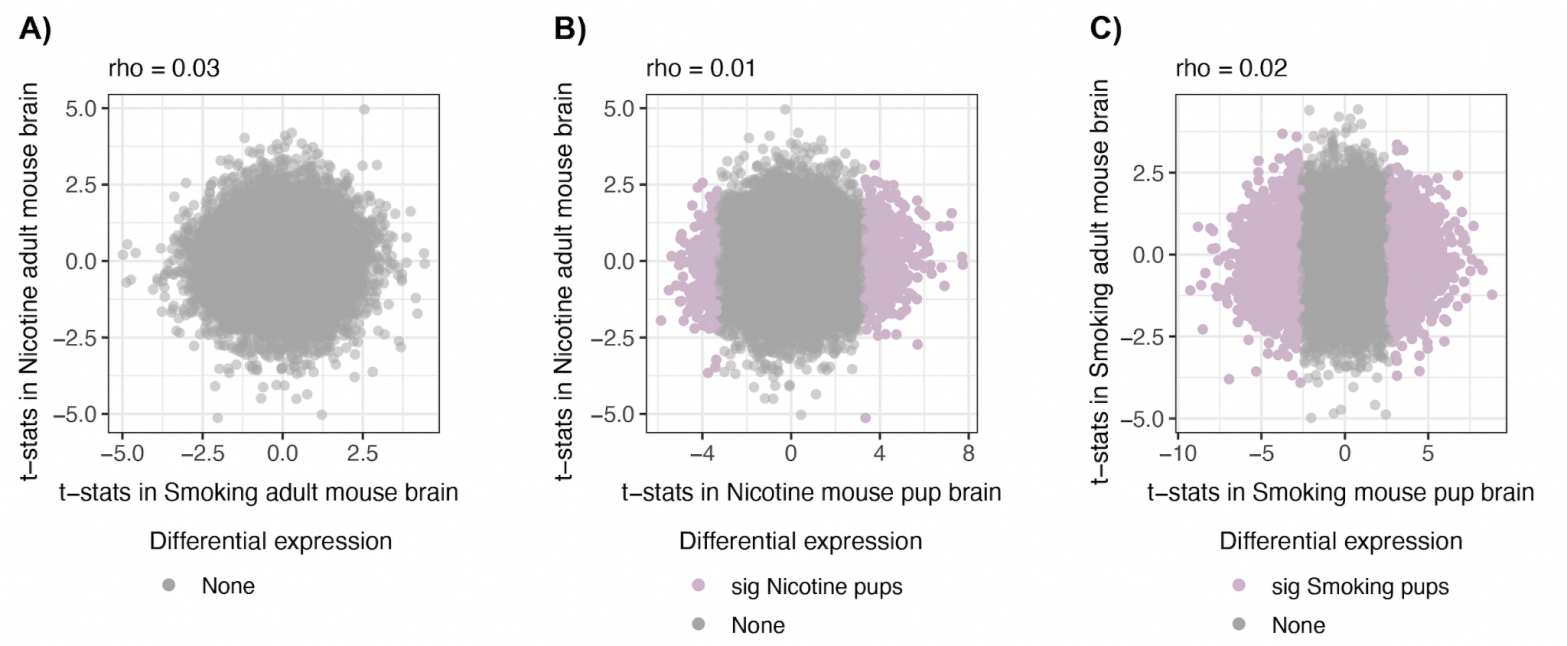
Differential gene expression signal on adult brain. Comparison of the moderated *t*-statistics of differential gene expression for **A)** nicotine administration vs smoking exposure in adult brain, and **B)** nicotine exposure and **C)** smoking exposure in adult vs pup brain. In light pink the DEGs in pup brain and in gray non-DEGs in any group; rho is the Spearman correlation coefficient. Related to Table S5.

To ascertain if the identified molecular impacts of nicotine and smoking exposure are specific to the developing brain, we compared those results to DGE findings for nicotine vs vehicle administration, and smoking exposure vs control in the adult brain (Fig. 1A). Both substances impacted differently on the gene expression in the adult brain (rho=0.03, Fig. 4A) but not significantly (0 DEGs at FDR<0.05, Fig. S13A,B), and the individual effects of each of these two substances were variable between adult vs pup brain (rho=0.01 and 0.02 for nicotine and smoking exposure, respectively, Fig. 4B,C; Table S5).

We extracted RNA from blood samples of the smoking-exposed adult dams and controls (Fig. 1A) to evaluate if brain-level transcriptomic changes caused by smoking exposure can be read out in blood. We performed DGE for smoking exposure vs control in blood but no DEGs were found (Fig. S13C) and the effects of cigarette smoke in blood and brain of adults at the gene level were uncorrelated (rho=−0.01, Fig. S21A, Table S5). Nevertheless, 37 (4.8%) of the 772 genes in adult brain with nominal differences (p<0.05) for smoking exposure vs control also had nominal differences in adult blood for smoking exposure (Fig. S21A, Table S5). And 3% of the smoking exposure-associated DEGs in pup brain replicated in smoking-exposed adult blood (rho=−0.04, Fig. S21B, Table S5). We also identified *KCNN2*, a human gene downregulated for smoking exposure in prenatal human brain (FDR<0.1) (12), replicating in smoking-exposed mouse blood (rho=0.03, Fig. S21C, Table S16).

### Comparison of mouse transcriptomic changes with findings in human

In a previous study, the transcriptional impacts of prenatal and adult exposure to smoking on human prefrontal cortex were assessed using 33 prenatal and 207 adult, non-psychiatric postmortem brain samples, respectively. The smoking-exposed phenotype was defined by nicotine or cotinine detectability. MSDP was directly associated with differential expression of 14 genes (FDR<0.1; 16 smoking-exposed vs 17 unexposed prenatal tissue samples), whereas only 2 genes were significantly differentially expressed in adult samples (FDR<0.1; 57 active smokers vs 150 non-smokers) (12).

**Figure 5:**
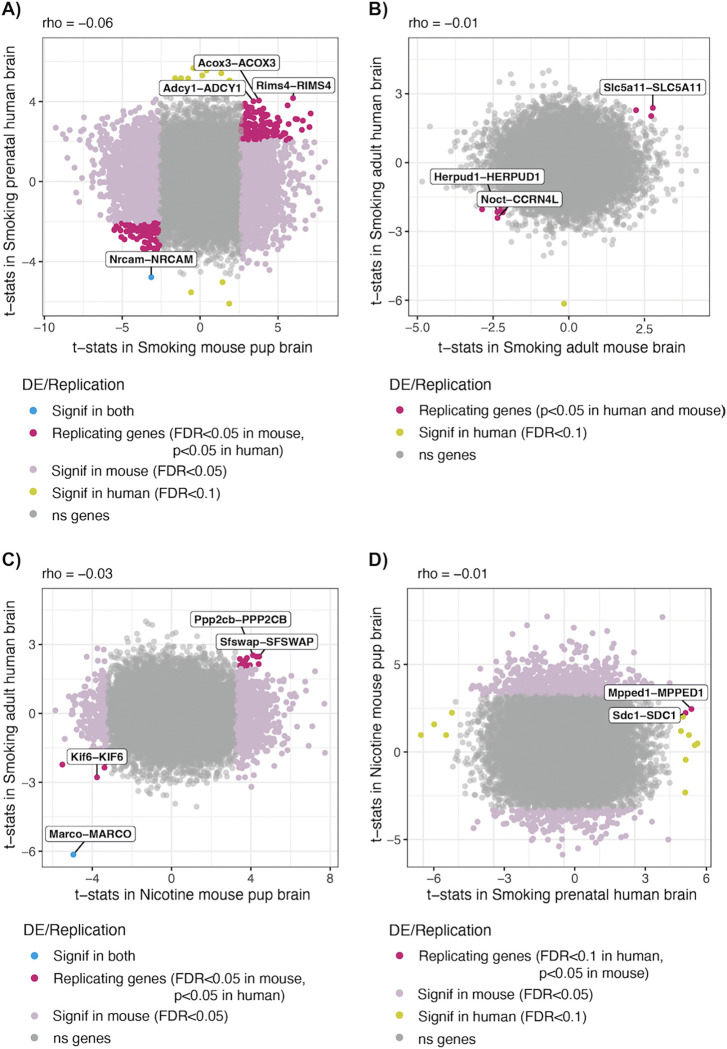
Differential gene expression signal for smoking exposure in mouse and human brain. Moderated *t*-statistics of the mouse genes for DE by (**A,B**) smoking exposure and (**C,D**) nicotine exposure in (**A,C,D**) pup and (**B**) adult mouse brain, compared against the moderated *t*-statistics of their human orthologs for smoking exposure in (**A,D**) prenatal and (**B,C**) adult human brain. In dark pink mouse (A-C) and human (D) brain genes that replicate in the other specie (with *p*-value<0.05 and same logFC sign); in light pink the genes that were DE in pup brain (FDR<0.05); in yellow the genes DE in human brain (FDR<0.1); in blue orthologous gene pairs that were DE in both species, and in gray non-DEGs in any specie. The gene pairs DE in both species, as well as the unique or the three replicating genes most significant in human are labeled with their mouse and human gene symbols. The Spearman correlation coefficient (rho) is shown above each plot. Related to Table S16.

We used the transcriptomic results of this study to assess the replicability of our mouse differential gene expression in human. Globally, we found uncorrelated effects of smoking exposure on mouse pup and adult brain compared against prenatal and adult postmortem human brain, respectively (Fig. 5A,B). Nevertheless, 267 out of 4,165 (6.41%) pup brain DEGs for smoking exposure replicated in the smoking-exposed human prenatal brain (rho=−0.06, Fig. 5A) and 9 out of 772 (1.17%) nominally DE genes (p<0.05) in the smoking-exposed adult mouse brain replicated in the smoking-exposed human adult brain (rho=−0.01, Fig. 5B, Table S16). In particular, *NRCAM* that encodes a cell adhesion protein required for cell-cell contacts in the brain, and its mouse ortholog, were significantly downregulated in smoking-exposed human prenatal and mouse pup brain, respectively (Fig. 5A). *MARCO* that encodes for a pattern recognition receptor (PRR) on immune cells, as well as its ortholog in mouse, were downregulated in the smoking-exposed human adult brain (12) and in the nicotine-exposed mouse pup brain, respectively (rho=−0.03, Fig. 5C). Moreover, the DEGs *MPPED1* and *SDC1* in the smoking-exposed prenatal human brain replicated in the nicotine-exposed mouse pup brain (rho=−0.01, Fig. 5D, Table S16).

Lastly, nicotine- and smoking-associated DEGs in the developing pup brain overlapped with candidate risk genes for tobacco use disorder (TUD), as identified in a genome-wide association study (GWAS) meta-analysis (25). Among the DEGs in offspring, human orthologs for *Trim35* and *Nr6a1* for nicotine exposure and *Chrna3*, *Rbm5*, *Sema3f*, and *Nfasc* for smoking exposure were associated with TUD and showed prenatal-specific expression, whereas the nicotine DEG *Vrk2* and the smoking DEGs *Drd2*, *Mtmr2*, and *Chrna3* were TUD-associated in the adult human brain. *Ip6k1* and *Cep57* were DE for both exposure experiments in pups and also associated with TUD. *Gmppb* and *P4htm* were two additional DEGs in smoking-exposed pup brain whose human orthologs were predicted to be affected in their expression by European single nucleotide polymorphisms (SNPs) TUD-associated in the human frontal cortex (Table S17).

## DISCUSSION

### Findings in pup and adult frontal cortex for nicotine and smoking exposure

This study interrogated transcriptomic effects of gestational smoking and nicotine exposure to the mother’s brain as well as the developing brain of the offspring in the mouse. Similar to observations in the smoking-exposed prenatal and adult human brain (12), we saw a wide signature for DE in the pup brain compared to the adult mouse brain. Likewise, reduced similarities in gene expression differences in the mature and developing brain were noted. Both findings are consistent with a stronger response to early compared to adult exposure (26,27).

Moreover, the effect of prenatal smoking exposure was more widespread than that of nicotine alone (4,165 vs 1,010 smoking-exposed and nicotine-exposed DEGs, respectively). This difference could be partly attributable to the larger number of samples used to model smoking exposure, but is also likely due to the composition of cigarette smoke, which contains >7,000 different chemicals besides nicotine (5). Therefore, although the overlap between genes affected by cigarette smoke and nicotine exposure was predictable, their effects were substantially different. Indeed, DEGs that were regulated in opposite directions in one and the other exposure reveal the differential impact on the same genes by nicotine alone and when interacting with thousands of other compounds present in the cigarette smoke. In addition, we cannot rule out differences in housing between the two experiments. Experiments were conducted in different facilities, and minor differences in standard housing, feed, or caging could contribute to differences across cohorts. Consequently, pups that were born to differently treated mice can also show experiment-dependent changes in gene expression.

Prenatal nicotine exposure significantly upregulated expression of *Foxn3* and *Arrdc3*. The former is essential for mice craniofacial development (28) and is associated with addictive substance use and compulsive behaviors in humans (29), whereas the second encodes an alpha-arrestin associated with neuroprotection in Parkinson’s disease (30) and acts as a regulator of locomotion (31), which agrees with previous results showing that PNE increases locomotor activity in mice (32,33). However, *Arrdc3*’s role in brain development remains to be explored. PNE also caused the downregulation of *Senp8*, which is involved in neural development (34), and *Coa4* which encodes a cytochrome *c* oxidase (COX) assembly factor whose downregulation may be linked to Leigh Syndrome or other related neurological disorders (35,36).

For maternal smoking during pregnancy, *Top2a* was the most significantly affected gene and was downregulated. This gene encodes for the DNA topoisomerase II alpha that regulates pluripotency and differentiation of embryonic stem cells (ESCs) (37). It has been demonstrated that maternal exposure to cigarette smoke components, such as metabolites of benzene, cause the transformation of the *Top2a* product into dangerous “molecular scissors” that fragment the genome and damage DNA in developing embryos (38). And it has been shown that the prenatal inhibition of *Top2a* causes postnatal autism-related behavioral defects in mice (39). *Tpx2* was the second most downregulated gene by prenatal smoking exposure and it plays crucial functions in the division, positioning, and fate of neural stem cells during mouse brain development (40). *AB041806* is a lncRNA gene and was the most upregulated after smoking exposure in pups; it is expressed in the CNS but the existence of its encoded protein has not been experimentally proven and its involvement in brain function is not known yet (41,42). The second most upregulated gene for prenatal smoking exposure was *Mt2*, which encodes a metallothionein (MT), a metal-binding protein that acts as an antioxidant and whose expression is known to be induced in the CNS as a response to brain damage (43). In fact, previous studies have identified *Mt2* as significantly upregulated in astrocytes after cerebral ischemic damage in mice (44) and following induced seizure attack in rats (45). In human MT genes have been found upregulated in astrocytes of patients with Alzheimer’s and Parkinson’s diseases (43,46,47).

Notably, genes that were upregulated after both PNE and MSDP play relevant roles in the cellular distribution and formation of protein complexes composed of several Sm proteins and the survival motor neuron (SMN) protein, such as *Strap*, *Snrpd3*, and *Snrpd*. The SMN-Sm complex is essential for spliceosomal small nuclear ribonucleoproteins (snRNPs) assembly in the cytoplasm for pre-mRNA splicing events and the highest levels of activity of this complex occur during embryonic and early postnatal development of the CNS (48). Other DEGs such as *Nsg1*, *Clstn1*, and *Rab4a* that were also upregulated in both experiments act in postsynaptic endosomes, which contribute to neural development regulation (49).

Genes uniquely upregulated by nicotine and not by smoking exposure were also identified, such as *Gsk3a*, *Ppp2r2b*, and *Ppp1cc* that are implicated in dopaminergic synapses, *Gnai3* and *Gnaq* that are related to synaptic long-term depression (LTD), as well as *Ppp2cb* which is involved in both pathways. These results are in alignment with previous findings reporting the nicotine interference in the dopamine neurotransmitter system development (11,50,51) and the nicotinic activity in LTD induction in rat and mouse brains (52–54). Besides, *Cplx2*, *Ykt6*, and *Cplx3* whose products participate in the SNARE complex were specifically upregulated by smoking but not by nicotine exposure, which could have relevant neurocognitive and behavioral implications, in support of the observed relationship between deficits in the SNARE protein SNAP-25 and maternal smoking with Attention Deficit Hyperactivity Disorder (ADHD) (33,55,56). Accordingly, the gestational exposure to smoking was associated with the upregulation of transcripts whose products act in the SNARE complex and are involved in vesicular transport, as well as transcripts of genes involved in Parkinson’s, Huntington’s, and prion-related diseases, such as *Casp3*, *Psma6*, and *Nduvf1*, placing MSDP as a potential not yet fully addressed environmental factor linked to the susceptibility or development of neurodegenerative disorders in offspring (57–59).

Together, the differential expression of these genes demonstrates a variable impact of cigarette smoke and nicotine on brain development and introduces potential long-term effects on the offspring related to neurodegenerative, neurodevelopmental, and substance use disorders. In the future, it will be informative to monitor behavioral and cognitive traits of the exposed newborn to gain insights into the postnatal effects related to prenatal nicotine and smoking exposure, as well as their underlying molecular processes including epigenetic modifications that may mediate the effect of nicotine and smoking exposure on gene expression.

### Blood vs brain molecular changes by smoking exposure in mice

Numerous epidemiological and toxicological studies have analyzed smoking effects in blood as an approach to establish brain effects. These studies either rely on the hypothesis that smoking components affect cardiovascular and brain health through the same responsive mechanisms, or that brain perturbations are, at least in part, a consequence of cardiovascular effects via circulation of pro-inflammatory mediators or ultrafine particulate matter that can reach the brain (60–62). Here we compared transcriptomic alterations caused by cigarette smoke exposure in adult brain and blood and found uncorrelated effects. Concordant with a previous investigation (63), this suggests that the study of cigarette smoke impact on brain cannot be addressed merely by the examination of blood samples.

Nonetheless, we found *Dusp14* downregulated in both smoking-exposed adult brain and blood (*p*-value<0.05 in both tissues). This gene regulates inflammation and oxidative stress and has been found downregulated in the infarcted area of mice after ischemic stroke (64), possibly linking tobacco exposure effects in brain and blood given that smoking is a well-recognized risk factor for stroke (62,65). *Syt13* was another gene downregulated in both tissues (*p*-value<0.05) with a role in neurotransmitter secretion by synaptic vesicles (66) but also recently characterized in human as a biomarker in lung adenocarcinoma (67), consistent with smoking exposure. A third adult brain gene replicating in blood was *Arhgef25*, also downregulated and whose human ortholog is expressed in brain vasculature (66). The upregulated DEG *Pde3b* in smoking-exposed pup brain also replicated in blood. Its expression is known to be increased after ischemic insult in the mouse brain (68) and accordingly its deletion/inhibition confers protection from ischemia/reperfusion (I/R) injury in mouse heart (69). The following most significant pup DEGs for smoking exposure replicating in blood were *Arhgap28* and *Slc39a6*, both downregulated and which need to be more widely studied in order to determine the relationship between their cigarette smoke effects on brain and blood. The downregulated DEG *KCNN2* in the smoking-exposed human prenatal brain (12) replicated in mouse blood. This gene is expressed in mouse brain and heart and several of its polymorphisms have been associated with cardiac tachyarrhythmias in human (70) and neurodevelopmental movement disorders and locomotor deficits in both humans and rodents (71,72). Moreover, its expression in brain is relevant for alcohol, nicotine, and drug addiction (73).

### Coincident molecular changes by smoking and nicotine exposure in mouse and human brain

Lastly, we explored to what extent our mouse results can be extrapolated to human using DGE results for smoking exposure in prenatal and adult human brain (12). An advantage of using mice to study the effects of prenatal and adult drug exposure is the ability to control experimental conditions that circumvents the confounding implications of human factors commonly coincident with drug use that also have impact on the brain, such as poor prenatal care and exposure to other substances (4), which makes it difficult to identify specific substance effects with certainty. However, the different gestation periods, routes of administration, pharmacokinetics, and correlation between transcriptomes of pups from the same litter, are some of the limitations of modeling these processes in animal models that hinder translatability to humans (4). In fact, our results indicate that the impacts of smoking exposure on gene expression in mouse and human brain are variable, which besides being explained by the inherent biological differences between species and experimental challenges modeling these processes, can be conceivable in terms of variations in the RNA-seq data processing steps and in the formal DGE analysis.

Nevertheless, DGE signal replicated between smoking/nicotine-exposed mouse brain and smoking-exposed human brain (12). The human gene *NRCAM* and its mouse ortholog were downregulated in the developing brain after cigarette smoke exposure (12). This gene encodes a neuronal cell adhesion protein with essential roles in axon growth and guidance and the formation of neural circuitry during brain development (74–80). *Nrcam*-null or deficient mice present autism-related behavioral and phenotypic alterations (75,77) and changes in its expression are associated with psychiatric disorders and drug addiction (74). *MARCO* was a downregulated gene in the smoking-exposed human adult brain (12) whose mouse ortholog was also downregulated in the nicotine-exposed pup brain, defining a gene expression change that is preserved regardless of species, age, and experiment setup. The product of this gene is a macrophage receptor with collagenous structure expressed in microglia involved in neuroinflammatory responses in neurodegenerative diseases (81,82). Its unknown involvement in neurodevelopment matches with its age-independent differential expression but it was not surprising to find it DE as it has been demonstrated that cigarette smoke exposure significantly decreases the expression of this gene in macrophages, which in turn leads to decreased pathogen clearance (83,84). Therefore, our results suggest nicotine and smoking can compromise brain immune function, as has been previously proposed (59,62).

Finally, finding DEGs in pup brain for both nicotine and smoking exposure, whose human orthologs are TUD-associated (25) with a matching brain region or developmental stage-specific expression, further suggests that MSDP and PNE can increase the likelihood of experimenting with drugs later in life, as has been extensively reported (85–92).

In summary, the present study revealed nicotine-specific and broader cigarette smoke transcriptomic effects on mouse brain development. The gene-level results were consistent and complemented with evidence at the transcript, exon, and exon-exon junction levels, finding DEGs and genes with other DE features with clear involvement in neurodevelopmental and behavioral processes. Also demonstrated were the variable effects of nicotine and cigarette smoke on the pup and adult mouse brain, as well as the non-extrapolable impact of tobacco smoke from mouse blood to brain, though, as presented, some genes subject to additional research could serve as biomarkers for smoking in these two tissues. Finally, these findings were supported by several human genes TUD-associated or affected by smoking in the prenatal and adult human prefrontal cortex that were also DE in the nicotine- and smoking-exposed pup brain. In conclusion, new insights into the genes and pathways implicated in the deleterious developmental effects of nicotine and cigarette smoke exposures during gestation were found and valuable data useful for ongoing research regarding the effects of MSDP and PNE were generated and publicly shared.

## Supplementary Material

Supplement 1

Supplement 2

## Data Availability

Unfiltered and normalized expression data generated and used in this project is available through the *smokingMouse* (94) Bioconductor data package, which also includes sample and feature-level data and the results from the DEA on human frontal cortex (12). Raw bulk RNA-seq files are available from the NCBI Sequence Read Archive (BioProject PRJNA1175674).

## Abbreviations

DE: differentially expressed or differential expression
DEA: differential expression analysis
DEE: differential exon expression
DEGs: differentially expressed genes
DGE: differential gene expression
DJE: differential exon-exon junction expression
DTE: differential transcript expression
FDR: false discovery rate
GO: Gene Ontology
GWAS: genome-wide association study
KEGG: Kyoto Encyclopedia of Genes and Genomes
MSDP: maternal smoking during pregnancy
P0: postnatal day 0
PNE: prenatal nicotine exposure
SNP: single nucleotide polymorphisms
TUD: tobacco use disorder
