# Supplementary material for: Molecular impact of nicotine and smoking exposure on the developing and adult mouse brain
Source: bioRxiv. 2024 Nov 5:2024.11.05.622149. Preprint. [Version 1] doi: 10.1101/2024.11.05.622149 (PMC11580964; doi:10.1101/2024.11.05.622149)
Supplement: Supplement 2 [file NIHPP2024.11.05.622149v1-supplement-2.pdf]

bioRxiv preprint doi: https://doi.org/10.1101/2024.11.05.622149; this version posted November 5, 2024. The copyright holder for this preprint (which was not certified by peer review) is the author/funder, who has granted bioRxiv a license to display the preprint in perpetuity. It is made available under aCC-BY 4.0 International license.

SUPPLEMENTARY MATERIAL

Supplementary Materials and Methods

Animals

Wild type male and female mice (C57BL/6J; stock # 000664, Jackson Laboratories) were purchased and used for timed breeding. 6 week old female mice were paired with male mice. Copulation plugs were checked daily and male mice were removed upon identification of plugs. Female mice were monitored for pregnancy, and separated upon pregnancy confirmation. Pups were euthanized by decapitation on the first day following birth, e.g. postnatal day 0 (P0). Pregnant dams were euthanized by decapitation and trunk blood was collected into a heparinized tube. Brains were rapidly extracted from the skull and the frontal cortex was dissected from the brain over wet ice on a steel block using a scalpel. Frontal cortical tissue was snap frozen in chilled 2-methylbutane. Samples were transferred to tubes and placed on dry ice and stored at -80°C until further processing for RNA extraction. All experiments and procedures were approved by the Johns Hopkins Animal Care and Use Committee and in accordance with the Guide for the Care and Use of Laboratory Animals.

Nicotine administration

Free-base(-)-nicotine (Sigma) was dissolved in normal saline. Nicotine (1.5mg/kg) or vehicle (saline) was administered to female dams (2X/daily - 8AM and 4PM). Administration started the week before mice were paired and continued until E17.

Smoking exposure

Pregnant dams were placed into a smoking chamber for 5 hours/day, 5 days/week starting one week before mice were paired for breeding and until the time of delivery. This chamber contains a smoking machine (Model TE-10, Teague Enterprises, Davis, CA) that burns 5 cigarettes (2R4F reference cigarettes (2.45 mg nicotine/cigarette; Tobacco Research Institute, University of Ky) at a time, taking 2 second duration puffs at a flow rate of 1.05 l/min, to provide a standard puff of 35 cm^3, providing a total of 8 puffs per minute. The machine is adjusted to produce side stream (89%) and mainstream smoke (11%). The chamber atmosphere is monitored to maintain total suspended particulate at 90 mg/m^3, and carbon monoxide at 350 ppm. Control pregnant dams were kept in a filtered air environment.

Tissue processing and RNA isolation and sequencing

Total RNA was extracted from samples using Trizol followed by purification with an RNeasy Micro kit (Qiagen). Paired-end strand-specific sequencing libraries were prepared and sequenced by Macrogen from 1ug total RNA using the TruSeq Stranded mRNA kit with ERCC

bioRxiv preprint doi: https://doi.org/10.1101/2024.11.05.622149; this version posted November 5, 2024. The copyright holder for this preprint (which was not certified by peer review) is the author/funder, who has granted bioRxiv a license to display the preprint in perpetuity. It is made available under aCC-BY 4.0 International license.

Spike in. Libraries were sequenced on an Illumina NovaSeq6000 S4, 150bp paired end. Output was targeted at 60M total reads (R1 30M and R2 30M) million 150-bp paired-end reads.

RNA-seq data processing

Expression quantification

Quality assessment of the sequencing reads and expression quantification at gene, exon, transcript, and exon-exon junction levels were performed running the RNA-seq processing pipeline *SPEAQeasy* version 6c1dab0 (13), using default settings, which involved alignment of reads to the Mus musculus genome from GENCODE M25 (95,96). As part of *SPEAQeasy, featureCounts* (19) was used for gene and exon quantification, using the `-O` argument for exon features in order to assign reads to all the overlapping exons. This setting has the drawback that it can inflate exon read counts. *RegTools* (97) was used for junction quantification and *Kallisto* (98) performed the pseudoalignment of the reads to the transcriptome.

Count normalization

Raw counts were normalized by sample library size calculating normalization factors with `calcNormFactors()` function from *edgeR* v3.43.7 (99) using the trimmed mean of M-values (TMM) method (100) for genes and exons, and the TMM with singleton pairing (TMMwsp) method for junctions. After rescaling library sizes, counts were transformed into counts per million (CPM) in a logarithmic scale with the *edgeR* `cpm()` function. For transcripts, transcripts per million (TPM) were log2-transformed after adding a 0.5 scaling factor (**Fig. S1**).

Feature filtering

Lowly-expressed genes, exons, and exon-exon junctions were filtered based on their counts across samples using `filterByExpr()` from *edgeR* v3.43.7 (99), which retains features that have a minimum of 15 reads across all samples and at least 10 reads in *n* or more samples, where *n* is 70% the size of the smallest sample group. Transcripts were filtered by defining a mean TPM expression cutoff of 0.28 with `expression_cutoff()` from *jaffelab* v0.99.32 (101) (**Fig. S1**).

RNA-seq data analysis

Exploratory data analysis

Quality control (QC) metrics of the samples were direct outputs of the *SPEAQeasy* pipeline (13) and additional ones were computed using `addPerCellQC()` from *scuttle* v1.9.4 (102) that can operate on counts at sample-level; all these metrics were calculated from raw counts of genes before normalization and filtering. These metrics were examined and compared separately for brain and blood samples, detecting large differences in the proportions of reads that mapped to

bioRxiv preprint doi: https://doi.org/10.1101/2024.11.05.622149; this version posted November 5, 2024. The copyright holder for this preprint (which was not certified by peer review) is the author/funder, who has granted bioRxiv a license to display the preprint in perpetuity. It is made available under aCC-BY 4.0 International license.

mitochondrial and ribosomal genes in adult and pup brain samples (**Fig. S2**), which led to age explaining a high percentage of gene expression variance in brain samples (**Fig. S3A**) and directed further separation of brain samples by age for downstream analyses.

Poor-quality samples were defined as those presenting lower outlier values for library size or number of detected genes, or higher outlier values for the percentage of either mitochondrial or ribosomal read counts. Values were considered outliers if they were 3 median-absolute-deviations away from the median, as defined by `isOutlier()` from *scater* v1.29.1 (102) (**Fig. S4**). After removing those samples, sources of gene, transcript, exon, and exon-exon junction expression variation in the samples were explored through dimensionality reduction analyses. Principal Component Analysis (PCA) revealed big differences in gene, transcript, and exon expression of brain samples from adult mice that were part of the nicotine and smoking exposure experiments, including both exposed and control samples each (**Fig. S5**, **Fig. S7A**), and exhibited 3 poor-quality samples that appeared isolated from the rest in PC plots (**Fig. S5**); these were manually filtered out. Similarly, three segregated poor-quality samples from pup brain were identified in PC plots, and sex appeared as a major driver of gene and exon expression variability (**Fig. S6**, **Fig. S7B**). In blood samples, pregnancy state slightly contributed to transcriptomic differences (**Fig. S3B**). Posterior to QC-based and manual sample filtering, 23 blood samples, 39 adult brain samples, and 130 pup brain samples were kept for downstream analyses (**Table S2**). Multidimensional scaling (MDS) analysis corroborated PCA results in the filtered adult and pup brain samples (**Fig. S8**, **Fig. S9**, **Fig. S10**).

In order to guide the selection of sample variables to include in the models for differential expression analysis, for each gene the percentage of expression variance explained by each individual explanatory variable was computed with `getVarianceExplained()` from *scater* v1.29.1 (102) (**Fig. S11**), as well as the fractions of variance explained (FVE) by each variable accounting for the joint contribution of all of them with `fitExtractVarPartModel()` from *variancePartition* v1.32.2 (103) (**Fig. S12**). In that way, QC metrics and biological variables such as pregnancy and sex, in adult and pup samples, respectively, were identified as major contributors to changes in the gene expression profiles. Pairs of highly correlated variables were recognized running a Canonical Correlation Analysis (CCA) with the *variancePartition* function `canCorPairs()` and only the one variable with the highest median FVE from each pair was added to the models (**Fig. S12**).

<u>Differential expression analyses</u>

Differentially expressed features for nicotine vs vehicle exposure/administration, and smoking exposure vs control were identified in pup brain, and adult brain and blood by defining the following models:

- For smoking exposure vs control in adult blood, and both nicotine vs vehicle administration and smoking exposure vs control in adult brain (analysis only at the gene level):
$$y_{ij} = \beta_{0i} + \beta_{1i}Group_j + \beta_{2i}Pregnancy_j + \beta_{3i}plate_j + \beta_{4i}flowcell_j + \beta_{5i}rRNA\_rate_j + \beta_{6i}totalAssignedGene_j$$

bioRxiv preprint doi: https://doi.org/10.1101/2024.11.05.622149; this version posted November 5, 2024. The copyright holder for this preprint (which was not certified by peer review) is the author/funder, who has granted bioRxiv a license to display the preprint in perpetuity. It is made available under aCC-BY 4.0 International license.

$$+ \beta_{7i}ERCCsumLogErr_j + \beta_{8i}overallMapRate_j + \varepsilon_{ij}$$

- For nicotine vs vehicle exposure, and smoking exposure vs control in pup brain (analysis at the four levels of expression features):

$$y_{ij} = \beta_{0i} + \beta_{1i}Group_j + \beta_{2i}Sex + \beta_{3i}plate_j + \beta_{4i}flowcell_j + \beta_{5i}rRNA\_rate_j + \beta_{6i}totalAssignedGene_j + \beta_{7i}ERCCsumLogErr_j + \beta_{8i}overallMapRate_j + \beta_{9i}mitoRate_j + \varepsilon_{ij}$$

Where y_{ij} denotes the expression of the ith feature in the jth sample, modeled by the selected covariates (see **Table S18** for their description) plus an error term ε_{ij}.

We applied an empirical Bayes analysis pipeline with *limma* v3.57.6 (14) for differential expression analysis. For gene, transcript, and exon counts, `voom()` was used as a first step to estimate inverse variance weights for each expression observation based on the mean-variance trend of the data to adjust for count heteroscedasticity (**Fig. S13**). This function renormalized raw counts into log-CPM using the previously computed normalization factors and library sizes for the non-filtered datasets. The log-normalized counts and their associated weights, as well as log-TPM of transcripts, were subsequently entered into `lmFit()` to fit a linear model by weighted least squares for each feature and estimate the model coefficients. Then `eBayes()` was used to moderate the residual sample standard deviations of the transcriptomic features through an empirical Bayes model. Finally *p*-values of the resulting moderated *t*-statistics were adjusted for multiple testing with the Benjamini and Hochberg's (BH) method (104) to control the FDR using `topTable()` (**Fig. S13**). Only genes, transcripts, and exon-exon junctions with an FDR<0.05, as well as exons with an FDR<0.05 and |log2FC|>0.25, were considered differentially expressed.

Replication of mouse brain DE results in mouse blood or human brain was defined with an FDR<0.05 for pup brain/*p*-value<0.05 for adult brain, a *p*-value<0.05 in blood/human, and the same regulation directionality in both tissues/species. Replication of human brain DE in mouse blood/brain was defined with an FDR<0.1 in human, *p*-value<0.05 in mouse, and same regulation directionality in both. Note however that when contrasting mice and human, results of gene pairs (mouse-human orthologs), but not individual genes, are compared.

Differential gene expression visualization

The z-scores of the log-normalized counts of DEGs were computed to visualize their expression patterns in heat maps, agglomerating genes and samples by expression through complete-linkage hierarchical clustering using an euclidean distance measure.

Novel junction gene annotation

The nearest (overlapping) neighbor and closest downstream and upstream (non-overlapping) genes of DE novel junctions were found using the functions `nearest()`, `precede()` and `follow()`, respectively, from *IRanges* v2.36.0 (105).

bioRxiv preprint doi: https://doi.org/10.1101/2024.11.05.622149; this version posted November 5, 2024. The copyright holder for this preprint (which was not certified by peer review) is the author/funder, who has granted bioRxiv a license to display the preprint in perpetuity. It is made available under aCC-BY 4.0 International license.

Software

ggplot2 v3.4.4 (106), *R* version 4.3.0 (107), and *Bioconductor* version 3.18 (108) were used to perform all the analyses and visualize the results.

bioRxiv preprint doi: https://doi.org/10.1101/2024.11.05.622149; this version posted November 5, 2024. The copyright holder for this preprint (which was not certified by peer review) is the author/funder, who has granted bioRxiv a license to display the preprint in perpetuity. It is made available under aCC-BY 4.0 International license.

Supplementary Tables

Supplementary Table 1: Study design. Number of samples from each pair of sample-level variables. See **Table S18** for sample variable description.

Supplementary Table 2: Samples used for downstream analyses. Number of samples from each pair of sample-level variables after sample filtering based on QC metrics and PCA plots. See **Table S18** for sample variable description.

Supplementary Table 3: DEGs in the nicotine-exposed pup brain. Metadata of DEGs in the nicotine pup brain and their logFC, moderated *t*-stats, *p*-value, and adjusted *p*-value for DE. The statistics were computed with `topTable()` from *limma*; see its documentation for the definition of the variable names. Related to **Fig. 2A**.

Supplementary Table 4: DEGs in the smoking-exposed pup brain. Same as in **Table S3** but for DEGs in the smoking pup brain. Related to **Fig. 2B**.

Supplementary Table 5: Differential gene expression results for the complete gene dataset. Gene-level metadata and the logFC, moderated *t*-stats, *p*-value, and adjusted *p*-value for DE of each gene in the 5 experimental groups: nicotine vs vehicle exposure in pup brain, smoking exposure vs control in pup brain, nicotine vs vehicle administration in adult brain, smoking exposure vs control in adult brain, and smoking exposure vs control in adult blood. Also included are the replication results of the genes in mouse blood. The statistics were computed with `topTable()` from *limma*; see its documentation for the definition of the variable names. Related to **Fig. 2C, Fig. 4** and **Fig. S21A-B**.

Supplementary Table 6: Differentially expressed transcripts in the nicotine-exposed pup brain. Metadata of DE transcripts in the nicotine pup brain and their logFC, moderated *t*-stats, *p*-value, and adjusted *p*-value. The statistics were computed with `topTable()` from *limma*; see its documentation for the definition of the variable names.

Supplementary Table 7: Differentially expressed transcripts in the smoking-exposed pup brain. Same as in **Table S6** but for DE transcripts in the smoking pup brain.

Supplementary Table 8: Differential expression of transcripts vs genes for the nicotine experiment in pup brain. DE statistics (logFC, moderated *t*-stats, *p*-value, and adjusted *p*-value) for transcripts and their respective genes for nicotine exposure in pup brain, and if transcripts and genes were both or solely DE. Only transcripts of genes present in the gene dataset are shown. The statistics were computed with `topTable()` from *limma*; see its documentation for the definition of the variable names. Related to **Fig. 3A**.

Supplementary Table 9: Differential expression of transcripts vs genes for the smoking experiment in pup brain. Same as in **Table S8** but for the smoking experiment. Related to **Fig. 3A**.

Supplementary Table 10: Differentially expressed exons in the nicotine-exposed pup brain. Metadata of DE exons in the nicotine pup brain and their logFC, moderated *t*-stats, *p*-value, and adjusted *p*-value. The statistics were computed with `topTable()` from *limma*; see its documentation for the definition of the variable names.

Supplementary Table 11: Differentially expressed exons in the smoking-exposed pup brain. Same as in **Table S10** but for DE exons in the smoking pup brain.

bioRxiv preprint doi: https://doi.org/10.1101/2024.11.05.622149; this version posted November 5, 2024. The copyright holder for this preprint (which was not certified by peer review) is the author/funder, who has granted bioRxiv a license to display the preprint in perpetuity. It is made available under aCC-BY 4.0 International license.

Supplementary Table 12: Differential expression of exons vs genes for the nicotine experiment in pup brain. DE statistics (logFC, moderated *t*-stats, *p*-value, and adjusted *p*-value) for exons and their respective genes for nicotine exposure in pup brain, as well as if exons and genes were both DE or not. Only exons of genes present in the gene dataset are shown. The statistics were computed with `topTable()` from *limma*; see its documentation for the definition of the variable names. Related to **Fig. 3B**.

Supplementary Table 13: Differential expression of exons vs genes for the smoking experiment in pup brain. Same as in **Table S12** but for the smoking experiment. Related to **Fig. 3B**.

Supplementary Table 14: Differentially expressed exon-exon junctions in the nicotine-exposed pup brain. Metadata of DE exon-exon junctions in the nicotine pup brain, including for each:

- if both the donor and acceptor sites together are known and annotated in GENCODE M25 (`inGencode` variable);
- if the donor and acceptor sites are individually annotated in GENCODE M25 (`inGencodeStart` and `inGencodeEnd` variables, respectively);
- the junction class: *Novel* (if both start and end sites are unknown, also known as fully novel junctions), *InGen* (already annotated in GENCODE M25), *AltStartEnd* (if it has only one known site), or *ExonSkip* (with sites from non-successive exons, both known individually but not together), and
- if they are fusion junctions, meaning that they connect exons from different genes (`isFusion` variable).

Their logFC, moderated *t*-stats, *p*-value, and adjusted *p*-value are provided. These statistics were computed with `topTable()` from *limma*; see its documentation for the definition of the variable names.

Supplementary Table 15: Differentially expressed exon-exon junctions in the smoking-exposed pup brain. Same as in **Table S14** but for DE exon-exon junctions in the smoking pup brain.

Supplementary Table 16: Differential gene expression results for gene pairs of mouse-human orthologs. The logFC, moderated *t*-stats, *p*-value, and adjusted *p*-value of the human gene for smoking exposure in the prenatal and adult human brain, and of the corresponding mouse orthologous gene for the 5 experimental mice groups (as in **Table S5**), are presented. Only mouse genes with human ortholog(s) present in the human dataset from (12) are considered. The DE statistics were computed with `topTable()` from *limma*; see its documentation for the definition of the variable names. Related to **Fig. 5** and **Fig. S21C**.

Supplementary Table 17: Mouse DEGs in pup brain with human orthologs TUD-associated. Pup brain DEGs for the nicotine and smoking exposure with human orthologs that were the nearest genes of genome-wide significant (GWS) lead SNPs in loci associated with TUD, obtained from a multi-ancestral GWAS meta-analysis of TUD cases and controls in individuals from 8 cohorts (including UKBB), with European (EUR), African American (AA), and Latin American (LA) ancestry (*TUD-multi+UKBB* dataset), and from a within-ancestry GWAS meta-analysis in EUR individuals from 5 cohorts, including UKBB data (*TUD-EUR+UKBB* dataset). As well as human genes significantly associated with TUD in EUR individuals (*TUD-EUR-MAGMA* dataset); neurobiologically relevant target human genes associated with TUD (*TUD-EUR-H-MAGMA* dataset), especially expressed in prenatal (*TUD-EUR-H-MAGMA-prenatal* dataset) and adult brain (*TUD-EUR-H-MAGMA-adult* dataset); TUD-associated human genes whose expression is predicted to be affected by EUR-SNPs across multiple brain regions (*TUD-EUR-S-MultiXcan* dataset) and

bioRxiv preprint doi: https://doi.org/10.1101/2024.11.05.622149; this version posted November 5, 2024. The copyright holder for this preprint (which was not certified by peer review) is the author/funder, who has granted bioRxiv a license to display the preprint in perpetuity. It is made available under aCC-BY 4.0 International license.

in specific brain regions (*TUD-EUR-S-PrediXcan* dataset), including the frontal cortex (*TUD-EUR-S-PrediXcan-FC* dataset). See more details of these TUD-associated human genes in the original publication (25).

Supplementary Table 18: Dictionary of sample variables. Description of the sample variables used throughout this project.

Supplementary Table 19: Associated genes of fully novel DE exon-exon junctions in pup brain. Nearest, following, and preceding genes of the fully novel DE exon-exon junctions without assigned gene for the nicotine and smoking exposure in pup brain.

bioRxiv preprint doi: https://doi.org/10.1101/2024.11.05.622149; this version posted November 5, 2024. The copyright holder for this preprint (which was not certified by peer review) is the author/funder, who has granted bioRxiv a license to display the preprint in perpetuity. It is made available under aCC-BY 4.0 International license.

Supplementary Figures

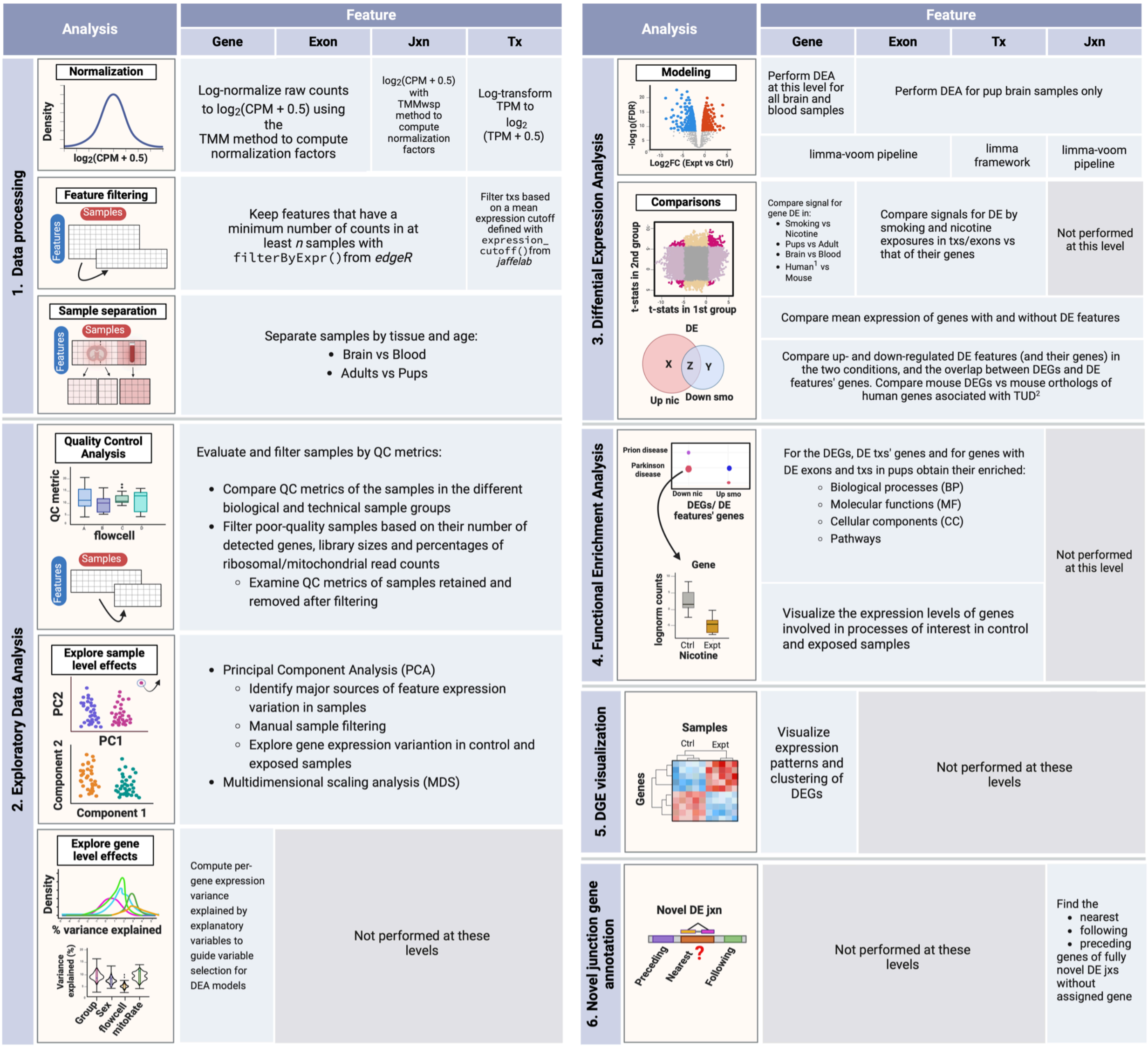

Supplementary Figure 1: Summary of analysis steps across gene expression feature levels. 1. Data processing: counts of genes, exons, and exon-exon junctions were normalized to CPM and log2-transformed; transcript expression values were only log2-transformed since they were already in TPM. Lowly-expressed features were removed using the indicated functions and samples were separated by tissue and age in order to create subsets of the data for downstream analyses. **2. Exploratory Data Analysis (EDA)**: QC metrics of the samples were examined and used to filter the poor quality ones. Sample level effects were explored through dimensionality reduction methods and segregated samples in PCA plots were removed from the datasets. Gene level effects were evaluated with analyses of variance partition. **3. Differential Expression Analysis (DEA)**: with the relevant variables identified in the previous steps, the DEA was performed at the gene level for nicotine and smoking exposure in adult and pup brain samples, and for smoking exposure in adult blood samples; DEA at the rest of the levels was performed for both exposures in pup brain only. DE signals of the genes in the different conditions, ages, tissues, and species ([1] using human results from Semick et al., 2020) were contrasted, as well as the DE signals of exons and transcripts vs those of their genes. Mean expression of DEGs and non-DEGs genes with and without DE features was also analyzed. Then, all resultant DEGs and DE features (and their genes) were compared by direction of regulation (up or down) between and within exposures (nicotine/smoking);

bioRxiv preprint doi: https://doi.org/10.1101/2024.11.05.622149; this version posted November 5, 2024. The copyright holder for this preprint (which was not certified by peer review) is the author/funder, who has granted bioRxiv a license to display the preprint in perpetuity. It is made available under aCC-BY 4.0 International license.

mouse DEGs were also compared against [2]human genes associated with TUD from Toikumo et al., 2023. **4. Functional Enrichment Analysis**: GO & KEGG terms significantly enriched in the clusters of DEGs and genes of DE transcripts and exons were obtained. **5. DGE visualization**: the log2-normalized expression of DEGs was represented in heat maps in order to distinguish the groups of up- and down-regulated genes. **6. Novel junction gene annotation**: for uncharacterized DE junctions with no annotated gene, their nearest, preceding, and following genes were determined. See **Supplementary Materials and Methods** for complete details. **Abbreviations:** Jxn: junction; Tx(s): transcript(s); CPM: counts per million; TPM: transcripts per million; TMM: Trimmed Mean of M-Values; TMMwsp: TMM with singleton pairing; QC: quality control; PC: principal component; DEA: differential expression analysis; DE: differential expression/differentially expressed; FC: fold-change; FDR: false discovery rate; DEGs: differentially expressed genes; TUD: tobacco use disorder; DGE: differential gene expression.

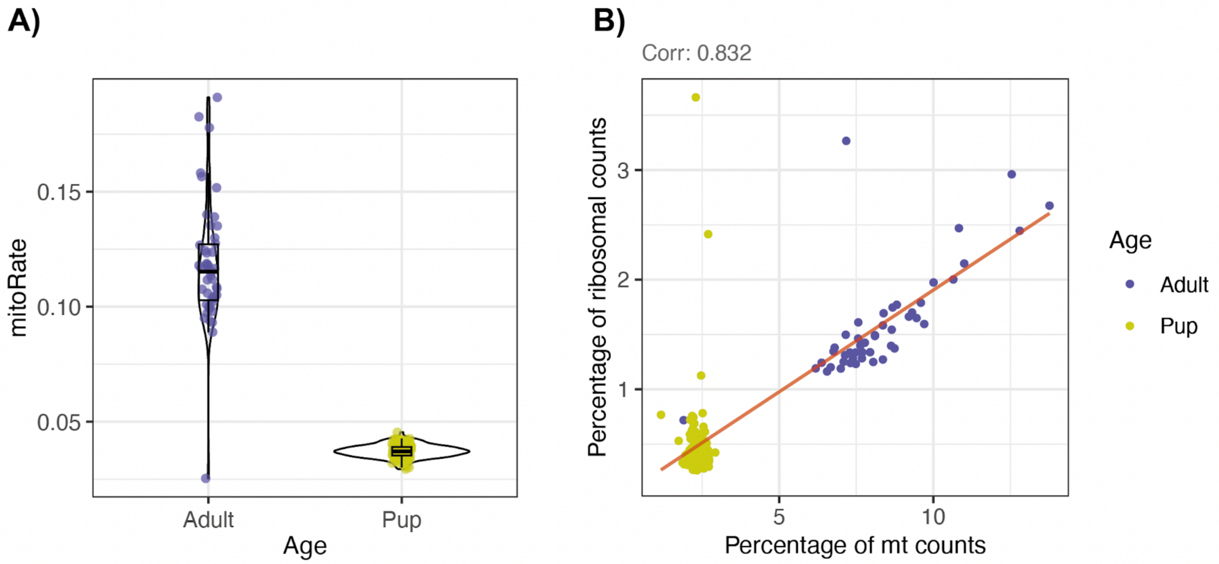

Supplementary Figure 2: Quality control metrics of adult and pup brain samples. **A)** The decimal fraction of reads that mapped to the mitochondrial chromosome of those that mapped at all in adult and pup brain samples. **B)** Percentage of sample counts from reads that were assigned to mitochondrial (mt) genes vs those that mapped to ribosomal genes, for each brain sample. Pearson correlation coefficient between these two QC metrics is shown above and the fitted linear regression line is shown in red.

bioRxiv preprint doi: https://doi.org/10.1101/2024.11.05.622149; this version posted November 5, 2024. The copyright holder for this preprint (which was not certified by peer review) is the author/funder, who has granted bioRxiv a license to display the preprint in perpetuity. It is made available under aCC-BY 4.0 International license.

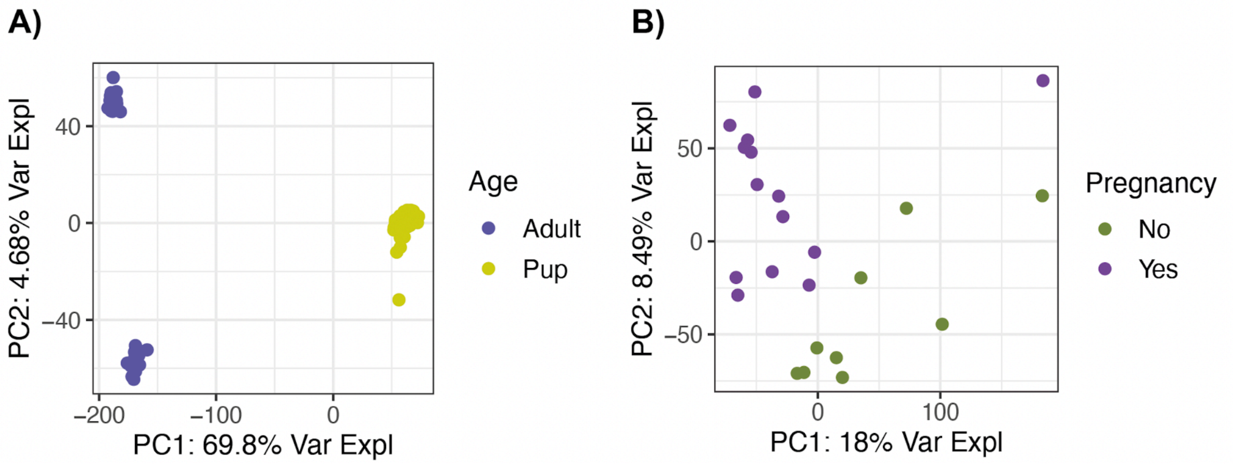

Supplementary Figure 3: Principal Component Analysis in brain and blood samples. Plots of principal component 1 (PC1) vs principal component 2 (PC2) for gene expression variation in **A)** brain and **B)** blood samples, separated by age and pregnancy state of mice, respectively. The percentage of variance explained by each PC is indicated in the axis labels.

bioRxiv preprint doi: https://doi.org/10.1101/2024.11.05.622149; this version posted November 5, 2024. The copyright holder for this preprint (which was not certified by peer review) is the author/funder, who has granted bioRxiv a license to display the preprint in perpetuity. It is made available under aCC-BY 4.0 International license.

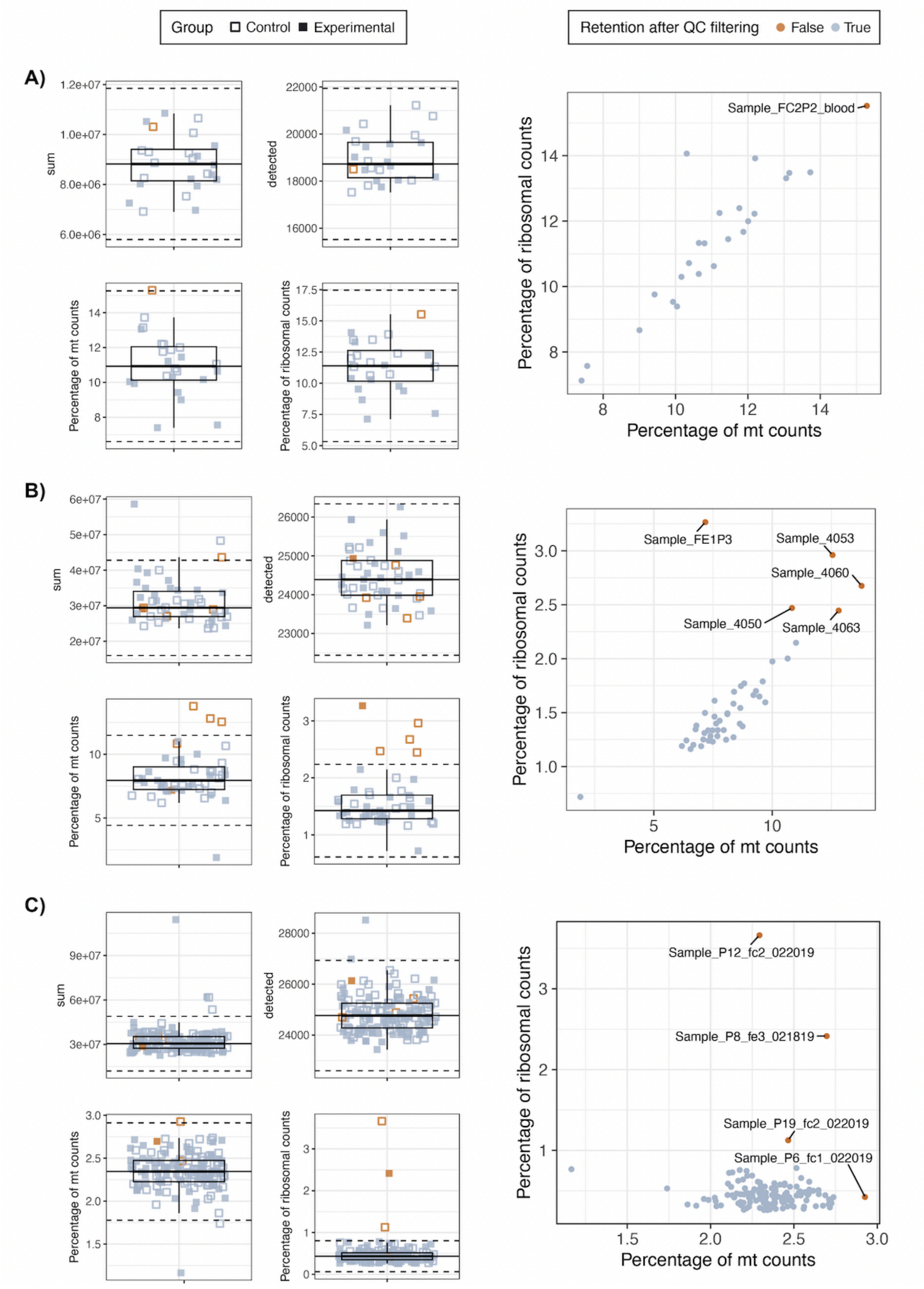

bioRxiv preprint doi: https://doi.org/10.1101/2024.11.05.622149; this version posted November 5, 2024. The copyright holder for this preprint (which was not certified by peer review) is the author/funder, who has granted bioRxiv a license to display the preprint in perpetuity. It is made available under aCC-BY 4.0 International license.

Supplementary Figure 4: Sample filtering by QC metrics. Left box plots show the library size (top left), the total number of detected genes (top right), the percentage of the total sample counts that correspond to reads mapping to mitochondrial (bottom left) and ribosomal (bottom right) genes in **A)** adult blood, **B)** adult brain and **C)** pup brain samples. In orange, the samples that were removed after sample filtering based on such metrics; in blue the ones that passed the filtering step. `Group` separates samples in smoking/nicotine-exposed and smoking/nicotine controls. Dotted lines are 3 median-absolute-deviations away from the median (solid line) and set the cutoff values to determine if samples were or not taken as outliers; lower outlier samples in library size or detected number of genes, and higher outlier samples in mitochondrial or ribosomal percentages were considered poor-quality and thus discarded. The scatter plots on the right show the same percentages of mitochondrial (mt) and ribosomal genes' read counts in all **A)** adult blood, **B)** adult brain and **C)** pup brain samples, labeling the filtered low-quality ones (in orange).

bioRxiv preprint doi: https://doi.org/10.1101/2024.11.05.622149; this version posted November 5, 2024. The copyright holder for this preprint (which was not certified by peer review) is the author/funder, who has granted bioRxiv a license to display the preprint in perpetuity. It is made available under aCC-BY 4.0 International license.

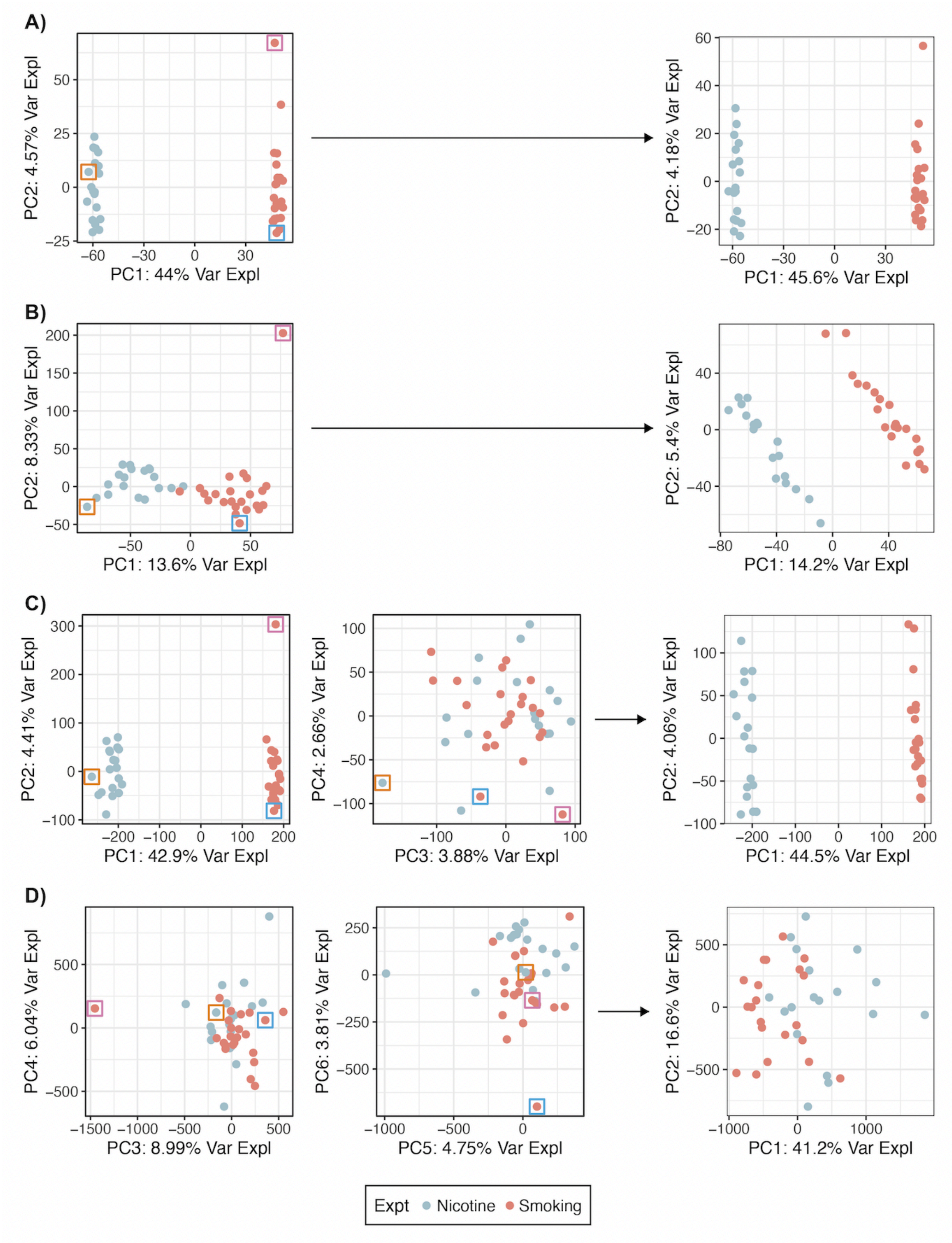

bioRxiv preprint doi: https://doi.org/10.1101/2024.11.05.622149; this version posted November 5, 2024. The copyright holder for this preprint (which was not certified by peer review) is the author/funder, who has granted bioRxiv a license to display the preprint in perpetuity. It is made available under aCC-BY 4.0 International license.

Supplementary Figure 5: Manual sample filtering of adult brain samples. Plots of Principal Components (PCs) for **A)** gene, **B)** transcript, **C)** exon and **D)** exon-exon junction expression variation in brain samples from adult mice. In blue the samples from the nicotine experiment and in orange from the smoking experiment (both experiments include exposed and control samples). Left and middle plots contain all samples that passed QC-based sample filtering (see **Fig. S4**); right plots resulted from removing poor-quality samples that appeared very far from the rest in PC plots, boxed in different colors; dots boxed in the same color correspond to the same sample.

- Pink boxed sample: turned out to be the sample with the highest proportion of rRNA counts.
- Orange boxed sample: is the sample with the highest proportion of reads that mapped to the mitochondrial chromosome; it has the highest percentages of mitochondrial and ribosomal genes' counts, the lowest proportion of reads assigned to genes and the minimum number of detected genes.
- Blue boxed sample: is the sample with the lowest decimal fraction of reads which successfully mapped to the reference genome and the smallest library size.

bioRxiv preprint doi: https://doi.org/10.1101/2024.11.05.622149; this version posted November 5, 2024. The copyright holder for this preprint (which was not certified by peer review) is the author/funder, who has granted bioRxiv a license to display the preprint in perpetuity. It is made available under aCC-BY 4.0 International license.

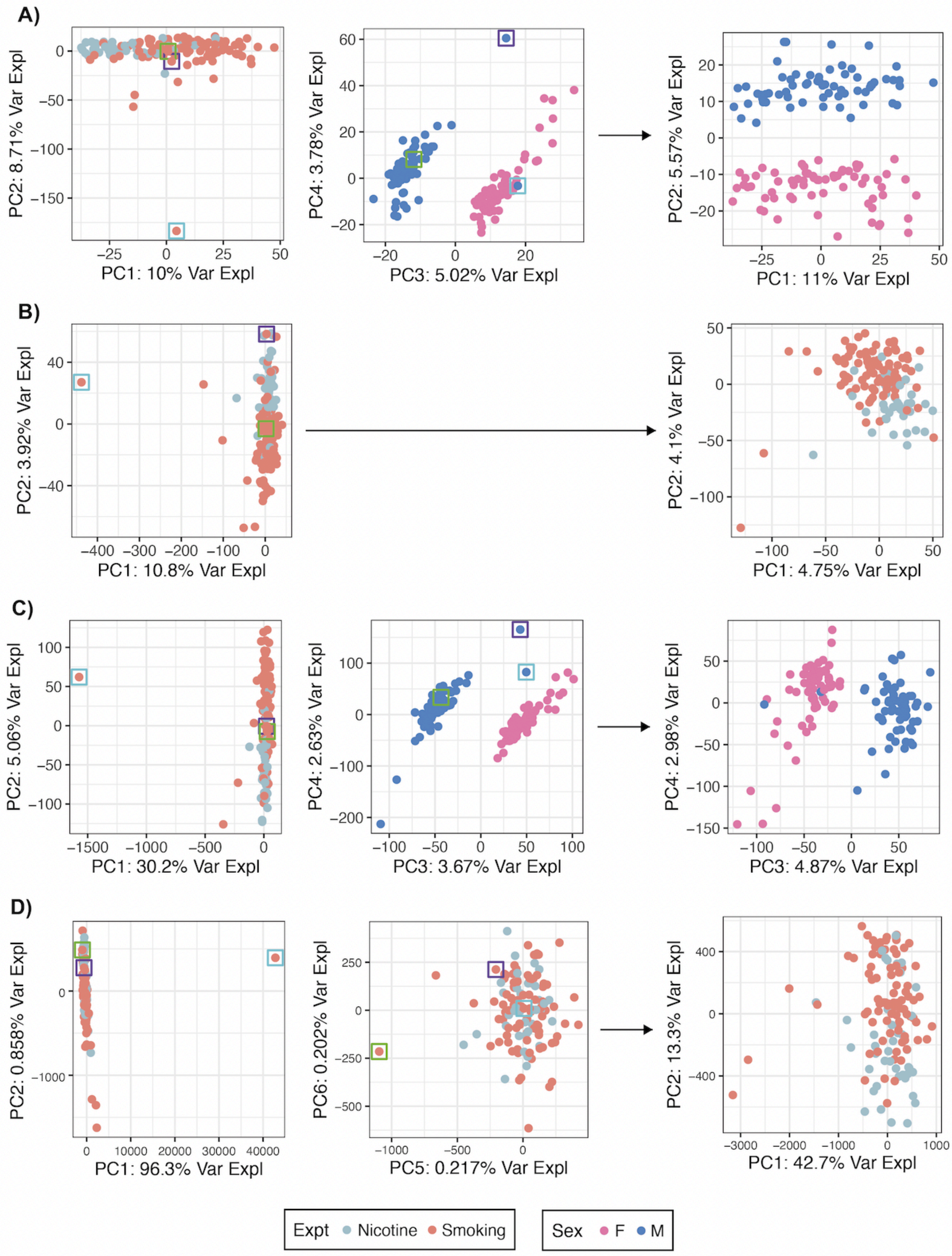

bioRxiv preprint doi: https://doi.org/10.1101/2024.11.05.622149; this version posted November 5, 2024. The copyright holder for this preprint (which was not certified by peer review) is the author/funder, who has granted bioRxiv a license to display the preprint in perpetuity. It is made available under aCC-BY 4.0 International license.

Supplementary Figure 6: Manual sample filtering of pup brain samples. Plots of principal components for **A)** gene, **B)** transcript, **C)** exon and **D)** exon-exon junction expression variation in brain samples from pups. Left and middle plots contain all samples that passed QC-based filtering (see **Fig. S4**); right plots resulted from removing segregated samples in PC plots, those are boxed in the same color for the same sample. 'Expt' separates samples by experiment (PNE and MSDP, both including exposed and control samples) and 'Sex' into female (F) and male (M).

- Blue boxed sample: is the sample with the lowest proportion of reads assigned to genes.
- Purple boxed sample: is the sample with the highest overall difference between the expected and the actual External Control Consortium (ERCC) RNA concentrations.
- Green boxed sample: is the sample with the lowest decimal fraction of reads which successfully mapped to the reference genome and the smallest library size.

bioRxiv preprint doi: https://doi.org/10.1101/2024.11.05.622149; this version posted November 5, 2024. The copyright holder for this preprint (which was not certified by peer review) is the author/funder, who has granted bioRxiv a license to display the preprint in perpetuity. It is made available under aCC-BY 4.0 International license.

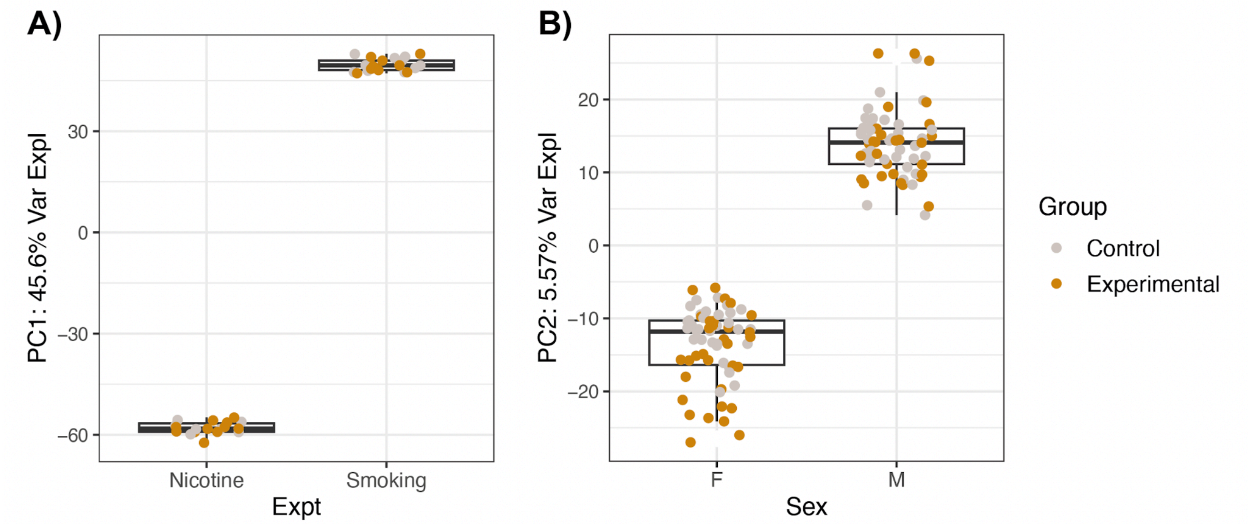

Supplementary Figure 7: Explore gene expression variation in experimental and control brain samples. Box plots of principal components for gene expression variation in **A)** adult brain samples from mice of nicotine and smoking experiments and **B)** brain samples from female and male pups. Gray dots correspond to control samples and the brown ones to nicotine/smoking exposed samples.

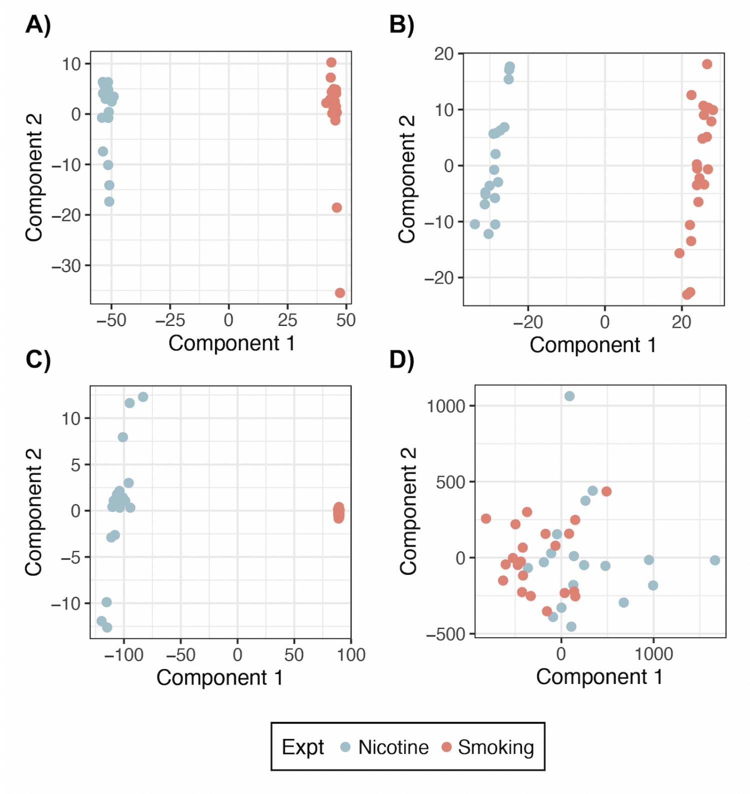

Supplementary Figure 8: Multidimensional scaling analysis in filtered adult brain samples. Component 1 vs Component 2 for **A)** gene, **B)** transcript, **C)** exon and **D)** exon-exon junction expression variation in adult brain samples from the nicotine and smoking experiments, including exposed and control samples. This analysis was done with samples that passed QC and manual sample filtering only (see **Fig. S4** and **Fig. S5**).

bioRxiv preprint doi: https://doi.org/10.1101/2024.11.05.622149; this version posted November 5, 2024. The copyright holder for this preprint (which was not certified by peer review) is the author/funder, who has granted bioRxiv a license to display the preprint in perpetuity. It is made available under aCC-BY 4.0 International license.

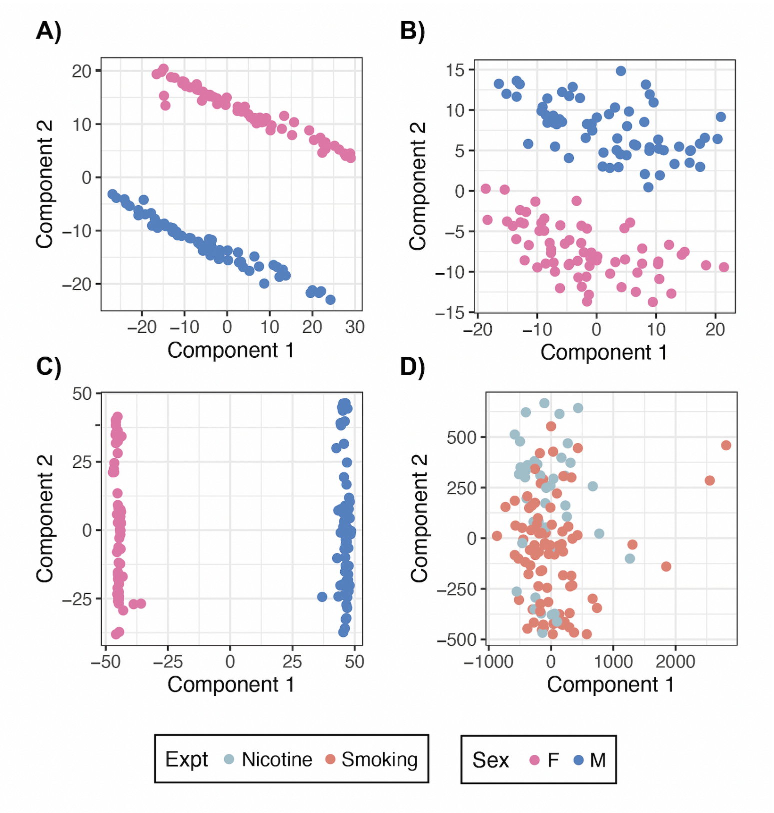

Supplementary Figure 9: Multidimensional scaling analysis in filtered pup brain samples. Component 1 vs Component 2 for **A)** gene, **B)** transcript, **C)** exon and **D)** exon-exon junction expression variation in pup brain samples that passed QC and manual sample filtering (see **Fig. S4** and **Fig. S6**). In **A)-C)** samples are separated by sex: females (F) and males (M); in **D)** by experiment, including exposed and control samples.

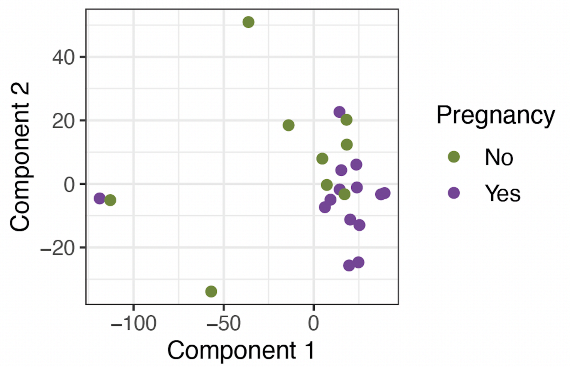

Supplementary Figure 10: Multidimensional scaling analysis in filtered blood samples. Component 1 vs Component 2 for gene expression variation in blood samples from pregnant and non-pregnant mice; these correspond to samples that passed QC sample filtering (see **Fig. S4**).

bioRxiv preprint doi: https://doi.org/10.1101/2024.11.05.622149; this version posted November 5, 2024. The copyright holder for this preprint (which was not certified by peer review) is the author/funder, who has granted bioRxiv a license to display the preprint in perpetuity. It is made available under aCC-BY 4.0 International license.

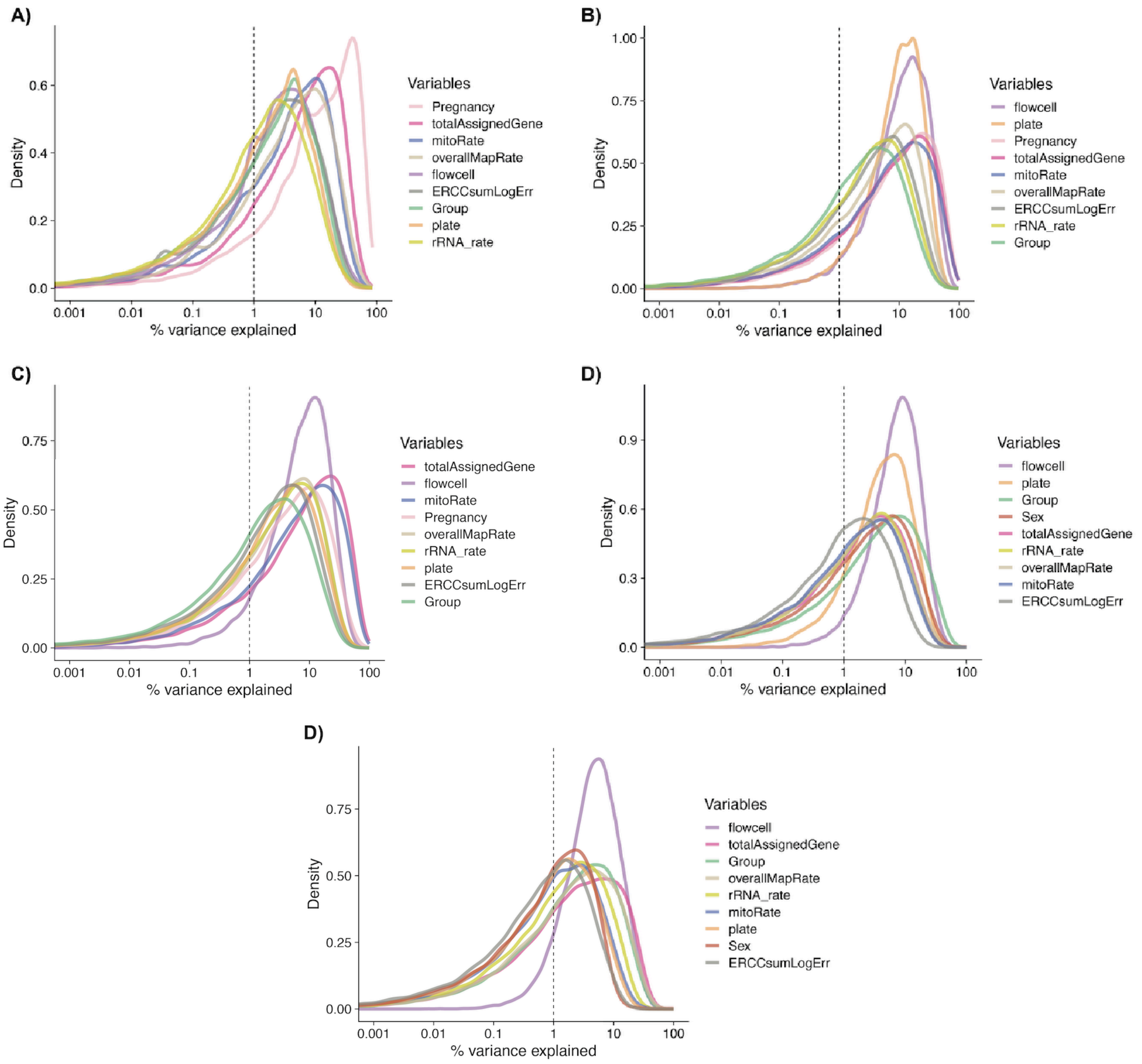

Supplementary Figure 11: Analysis of variance in gene expression explained by the explanatory sample variables. Density plots for the percentage of variance in the expression of each gene that is explained by each sample-level variable in **A)** blood samples, **B)** adult brain samples from the nicotine or **C)** smoking experiment and **D)** pup brain samples from the nicotine or **E)** smoking experiment. See **Table S18** for the description of the covariates.

bioRxiv preprint doi: https://doi.org/10.1101/2024.11.05.622149; this version posted November 5, 2024. The copyright holder for this preprint (which was not certified by peer review) is the author/funder, who has granted bioRxiv a license to display the preprint in perpetuity. It is made available under aCC-BY 4.0 International license.

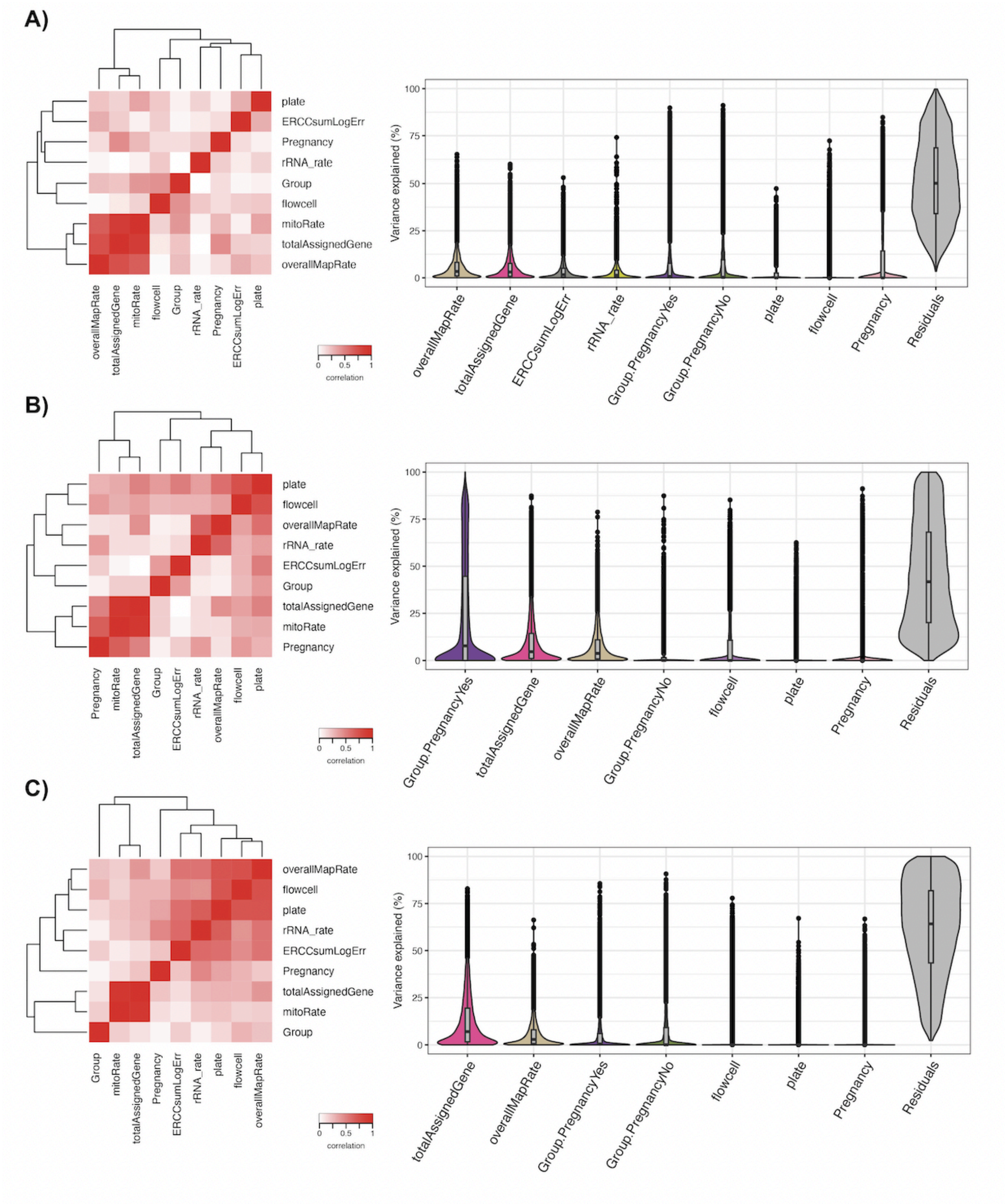

bioRxiv preprint doi: https://doi.org/10.1101/2024.11.05.622149; this version posted November 5, 2024. The copyright holder for this preprint (which was not certified by peer review) is the author/funder, who has granted bioRxiv a license to display the preprint in perpetuity. It is made available under aCC-BY 4.0 International license.

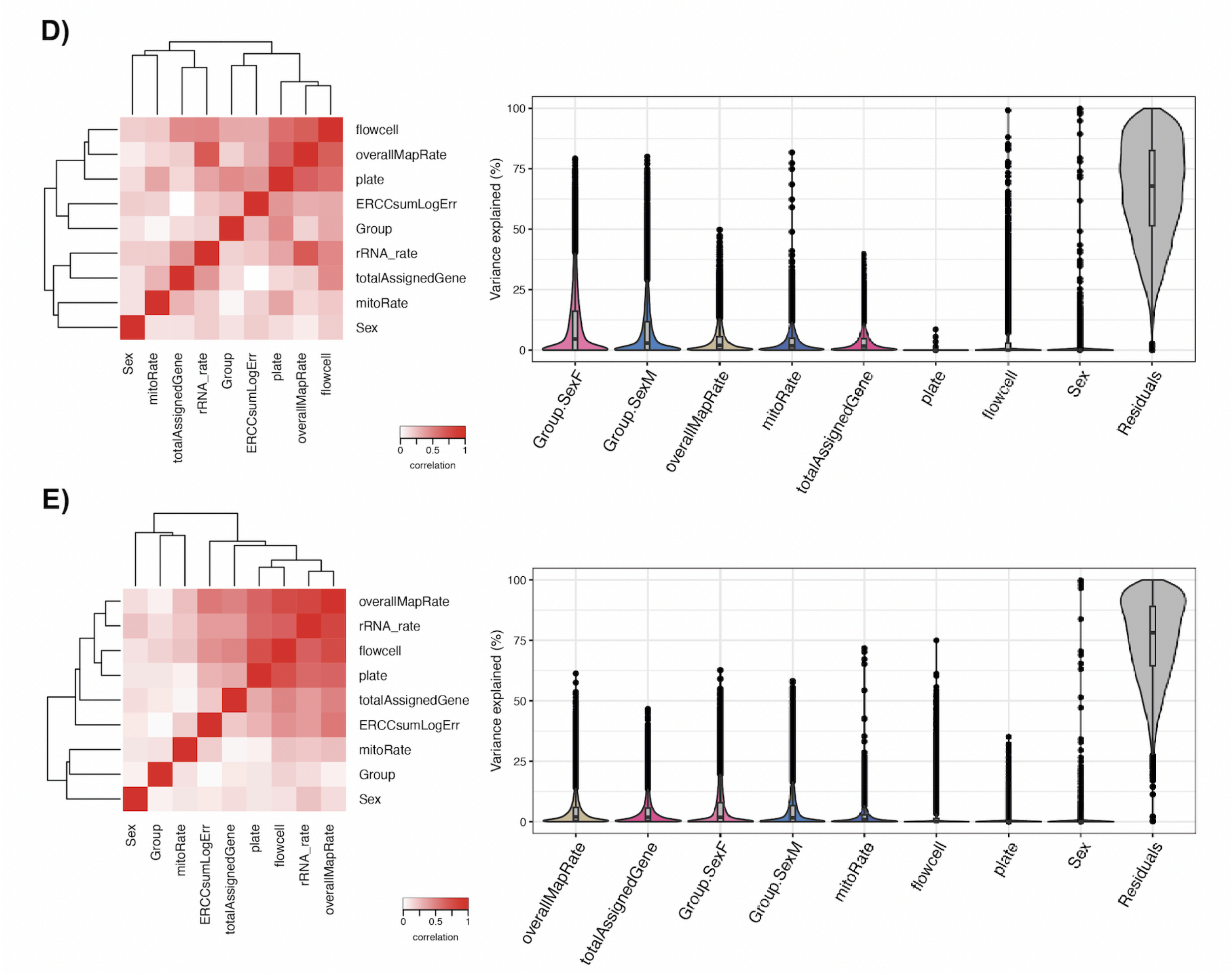

Supplementary Figure 12: Variance partition analysis. Left heat maps show the correlation (from 0 to 1) between each pair of sample variables, obtained through a Canonical Correlation Analysis (CCA). Violin plots show for each gene the percentages of variance in their expression levels that are explained by each variable in **A)** blood samples of the smoking experiment, **B)** adult brain samples of the nicotine experiment, **C)** adult brain samples of the smoking experiment, **D)** pup brain samples of the nicotine experiment, and **E)** pup brain samples of the smoking experiment. Variables are ordered by decreasing mean fraction of variance explained (FVE). Variables in the heat maps not present in the corresponding violin plots were highly correlated with any other variable with a higher median FVE and thus were not included in the models for DGE. `ERCCsumLogErr` and `rRNA_rate` were not considered in the brain samples because their scales differ considerably and therefore were not suitable for variance partition. Labels `Group:Pregnancy` in **A)-C)** and `Group:Sex` in **D)** and **E)** refer to the interaction of nicotine/smoking exposure (`Group`) with pregnancy and sex, respectively. See **Table S18** for the description of these sample variables. Residuals correspond to those fractions of gene expression variation that could not be attributed to any of the sample-level variables.

bioRxiv preprint doi: https://doi.org/10.1101/2024.11.05.622149; this version posted November 5, 2024. The copyright holder for this preprint (which was not certified by peer review) is the author/funder, who has granted bioRxiv a license to display the preprint in perpetuity. It is made available under aCC-BY 4.0 International license.

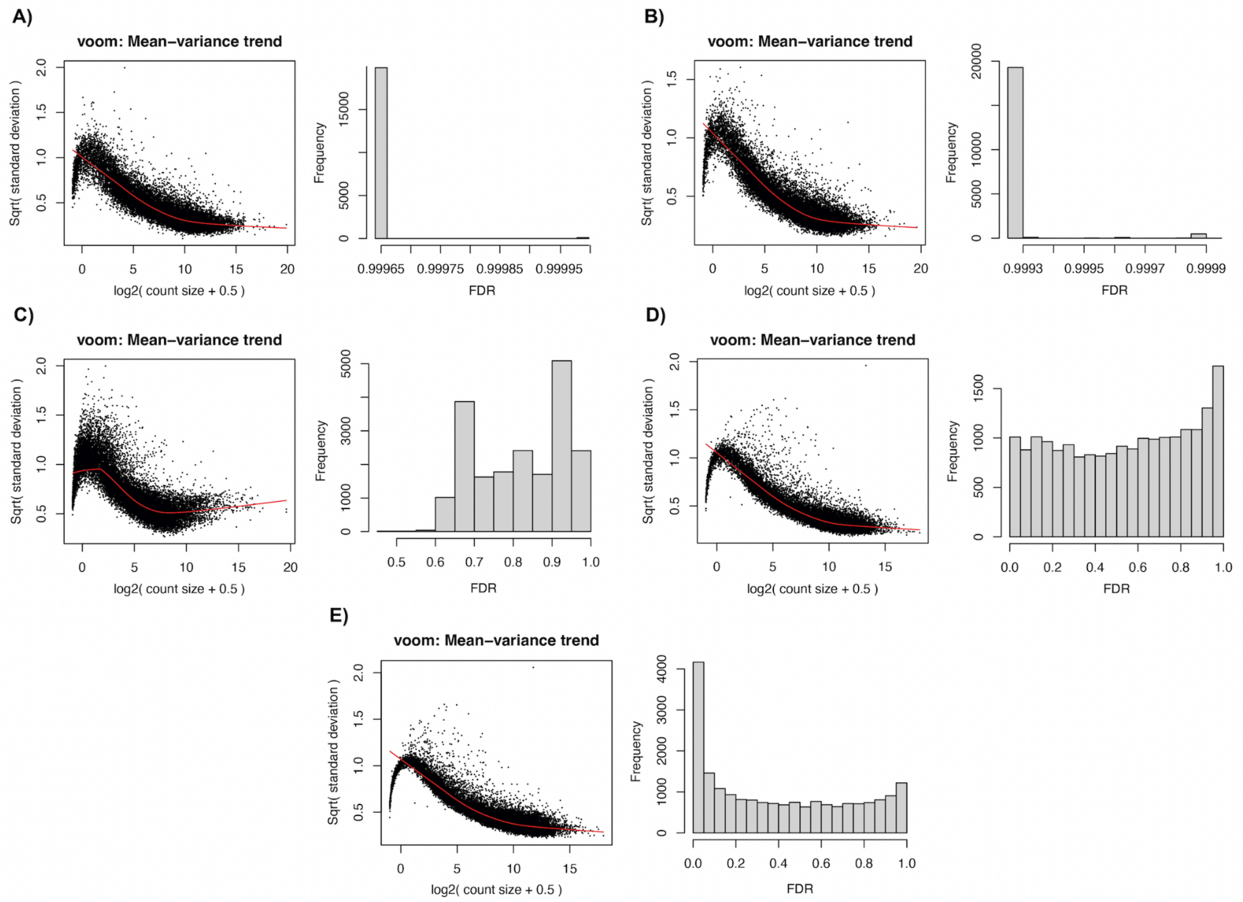

Supplementary Figure 13: **Results of differential gene expression analyses**. Left plots show for each gene their mean expression (in log2-counts) and the square-root of their residual standard deviations in **A)** adult brain samples of the nicotine experiment, **B)** adult brain samples of the smoking experiment, **C)** adult blood samples of the smoking experiment, **D)** pup brain samples of the nicotine experiment, and **E)** pup brain samples of the smoking experiment. Red line corresponds to the global mean-variance trend. Histograms present gene-wise FDR-adjusted *p*-values for differential expression in the same sample groups.

bioRxiv preprint doi: https://doi.org/10.1101/2024.11.05.622149; this version posted November 5, 2024. The copyright holder for this preprint (which was not certified by peer review) is the author/funder, who has granted bioRxiv a license to display the preprint in perpetuity. It is made available under aCC-BY 4.0 International license.

A)

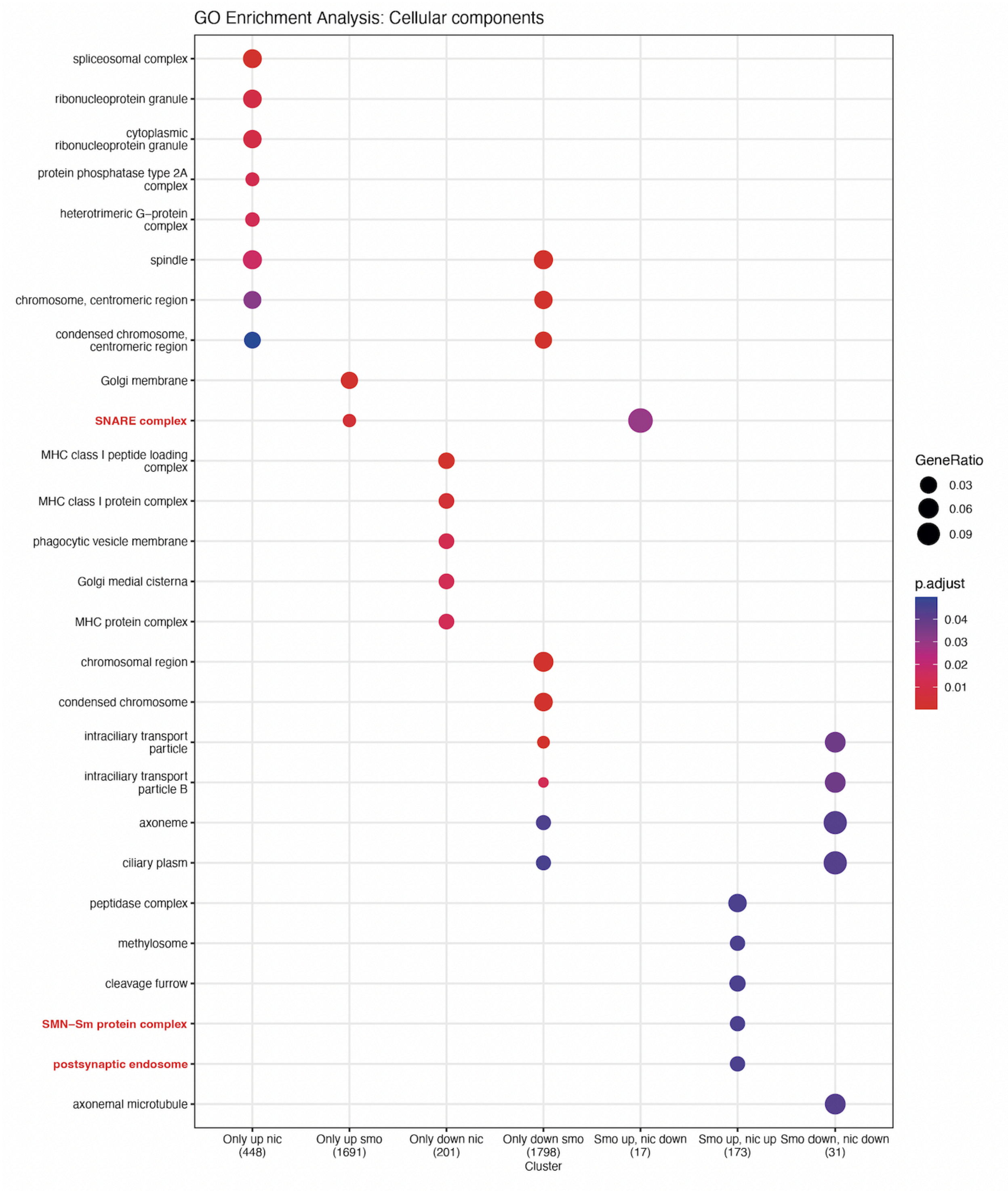

bioRxiv preprint doi: https://doi.org/10.1101/2024.11.05.622149; this version posted November 5, 2024. The copyright holder for this preprint (which was not certified by peer review) is the author/funder, who has granted bioRxiv a license to display the preprint in perpetuity. It is made available under aCC-BY 4.0 International license.

B)

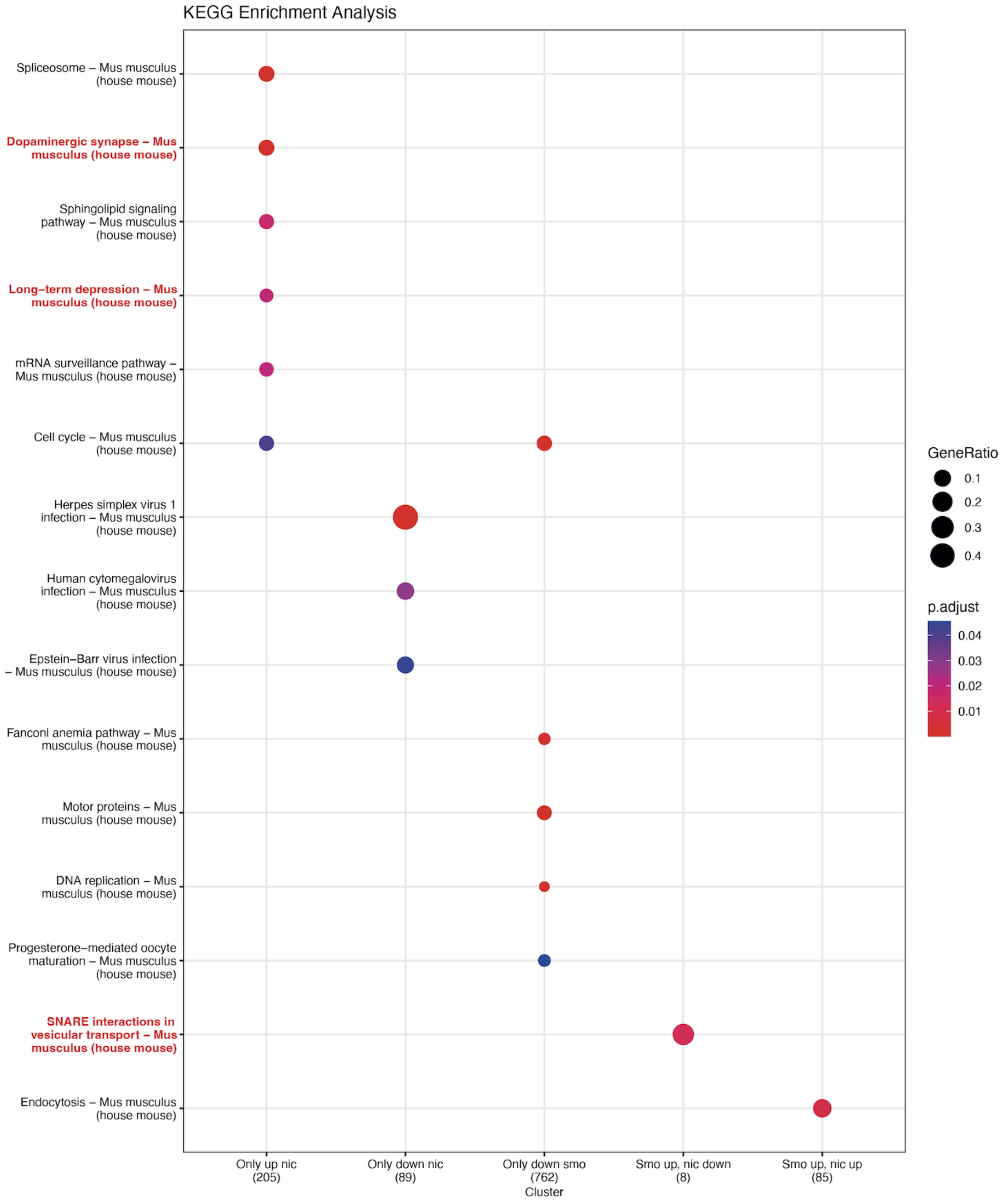

bioRxiv preprint doi: https://doi.org/10.1101/2024.11.05.622149; this version posted November 5, 2024. The copyright holder for this preprint (which was not certified by peer review) is the author/funder, who has granted bioRxiv a license to display the preprint in perpetuity. It is made available under aCC-BY 4.0 International license.

C)

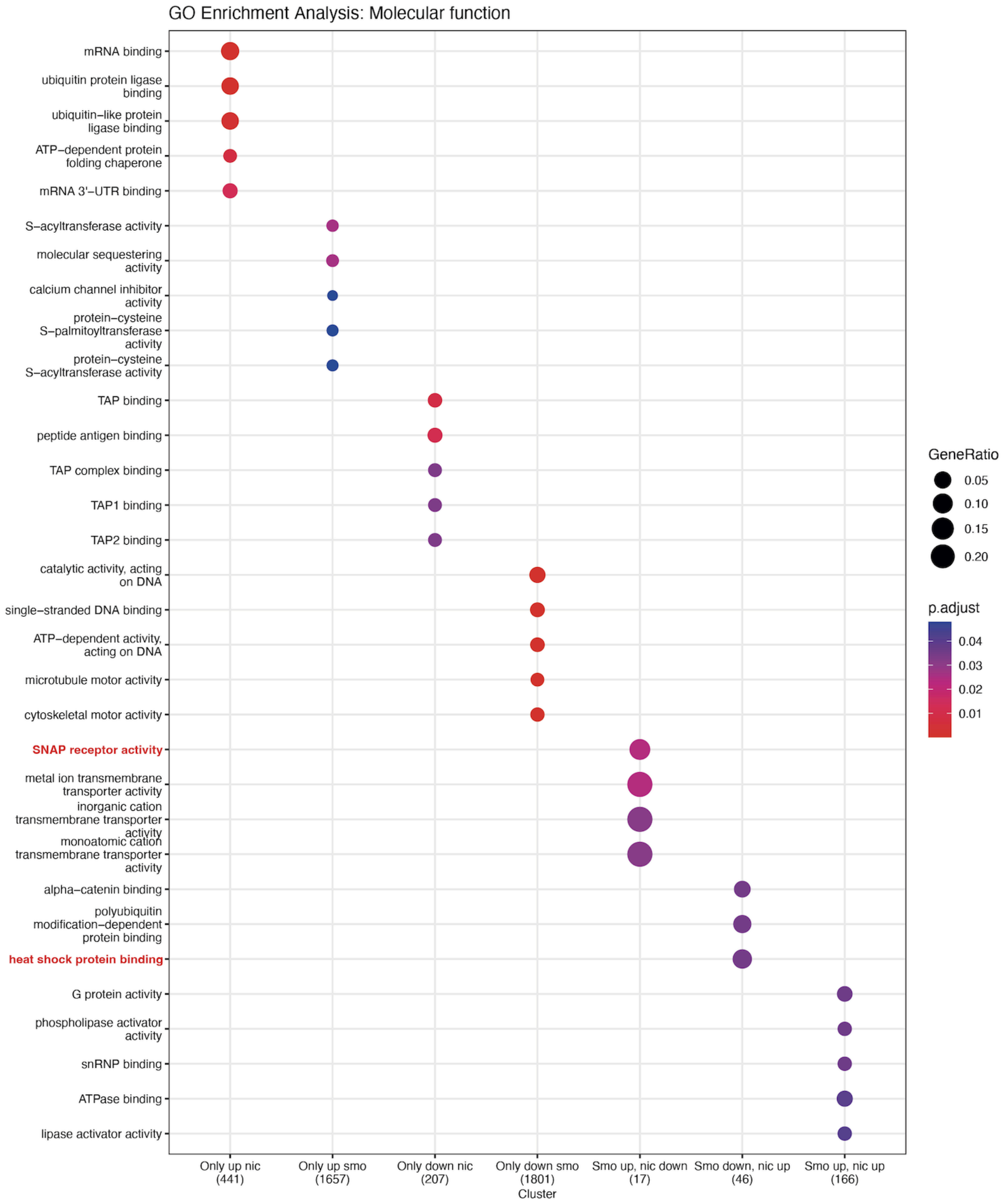

bioRxiv preprint doi: https://doi.org/10.1101/2024.11.05.622149; this version posted November 5, 2024. The copyright holder for this preprint (which was not certified by peer review) is the author/funder, who has granted bioRxiv a license to display the preprint in perpetuity. It is made available under aCC-BY 4.0 International license.

D)

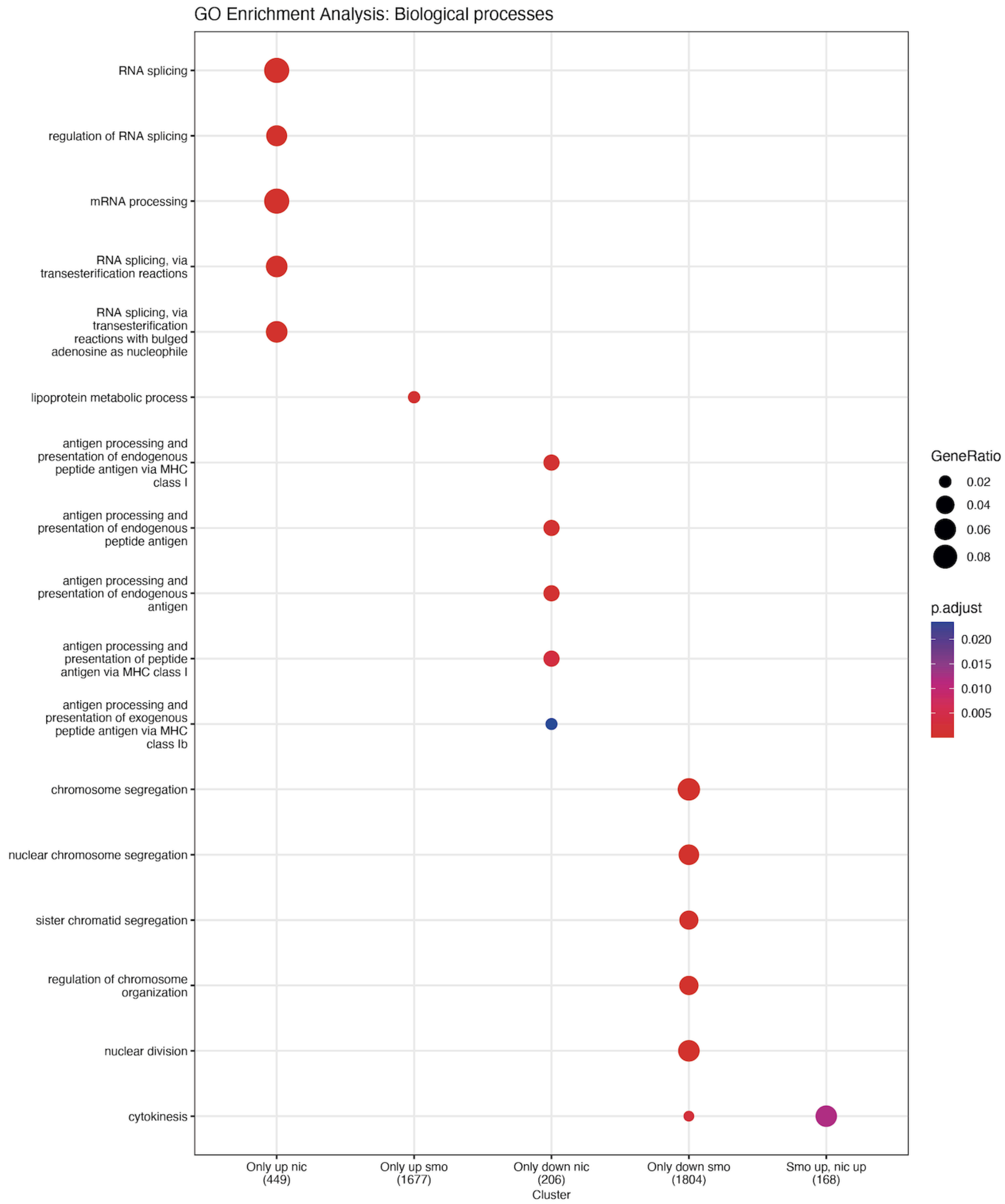

bioRxiv preprint doi: https://doi.org/10.1101/2024.11.05.622149; this version posted November 5, 2024. The copyright holder for this preprint (which was not certified by peer review) is the author/funder, who has granted bioRxiv a license to display the preprint in perpetuity. It is made available under aCC-BY 4.0 International license.

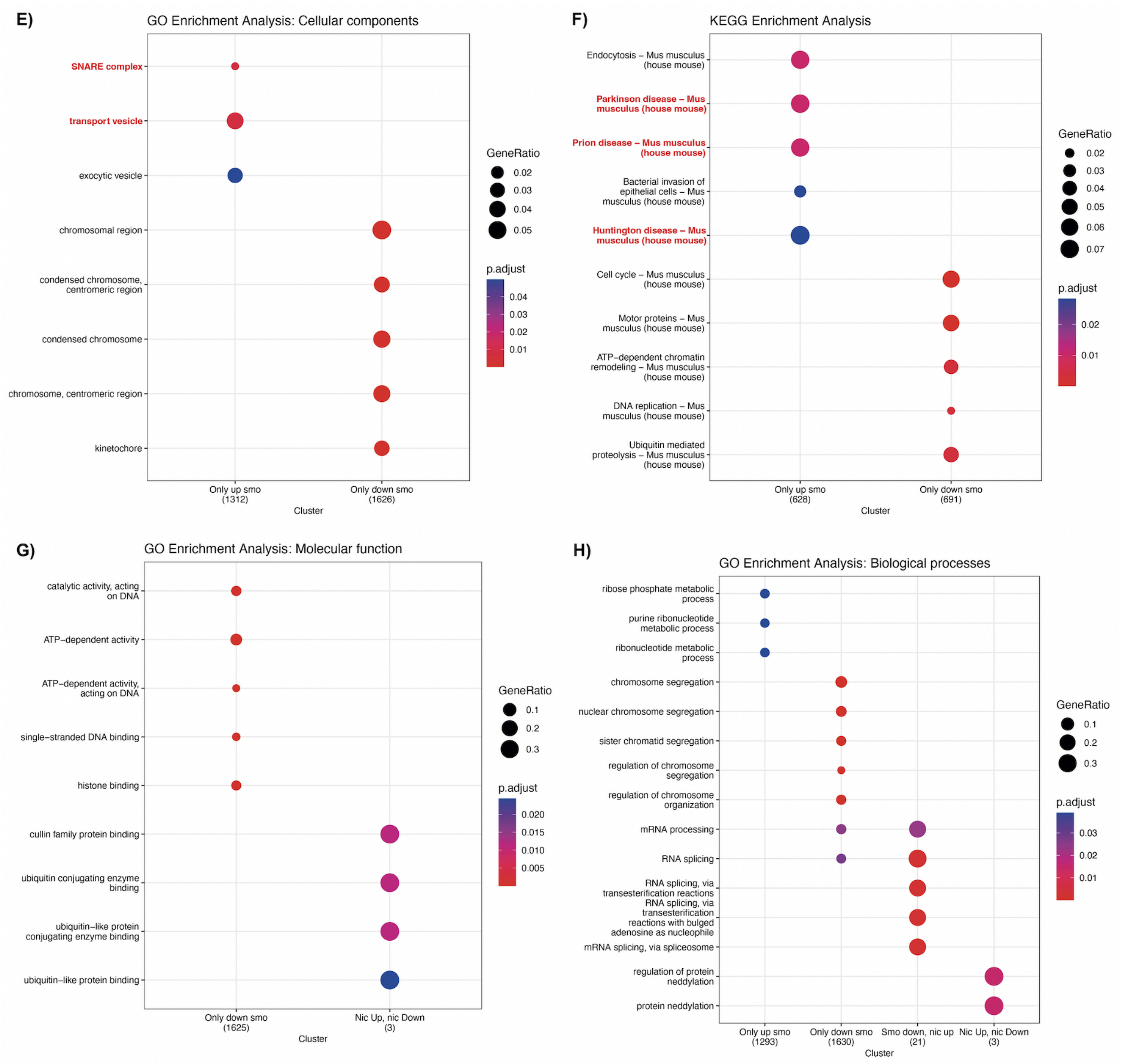

Supplementary Figure 14: Functional enrichment analysis for DEGs and DE transcripts' genes in pup brain. Cellular components (CC), pathways, molecular functions (MF) and biological processes (BP) significantly overrepresented (FDR-adjusted p-value<0.05) in the clusters of (**A-D**) DEGs and (**E-H**) genes with DE transcripts, indicated in the x-axis. Clusters without significant results are excluded; `up` and `down` labels stand for upregulated and downregulated, respectively, and `only` refers to genes that were significant (**A-D**) or had significant transcripts (**E-H**) in either the smoking (`smo`) or nicotine (`nic`) experiment but not in the other. Note that cluster numbers (in parentheses) correspond to the number of genes in the specified cluster that are annotated in at least one GO/KEGG term. Gene ratio is the number of genes in each cluster annotated in a term over the total in the respective cluster. Only the top 5 most significant enriched terms are reported per cluster, unless they share additional significant terms with other clusters. Terms of interest appear in red (see genes involved in each in **Fig. S15**). Related to **Fig. 2**.

bioRxiv preprint doi: https://doi.org/10.1101/2024.11.05.622149; this version posted November 5, 2024. The copyright holder for this preprint (which was not certified by peer review) is the author/funder, who has granted bioRxiv a license to display the preprint in perpetuity. It is made available under aCC-BY 4.0 International license.

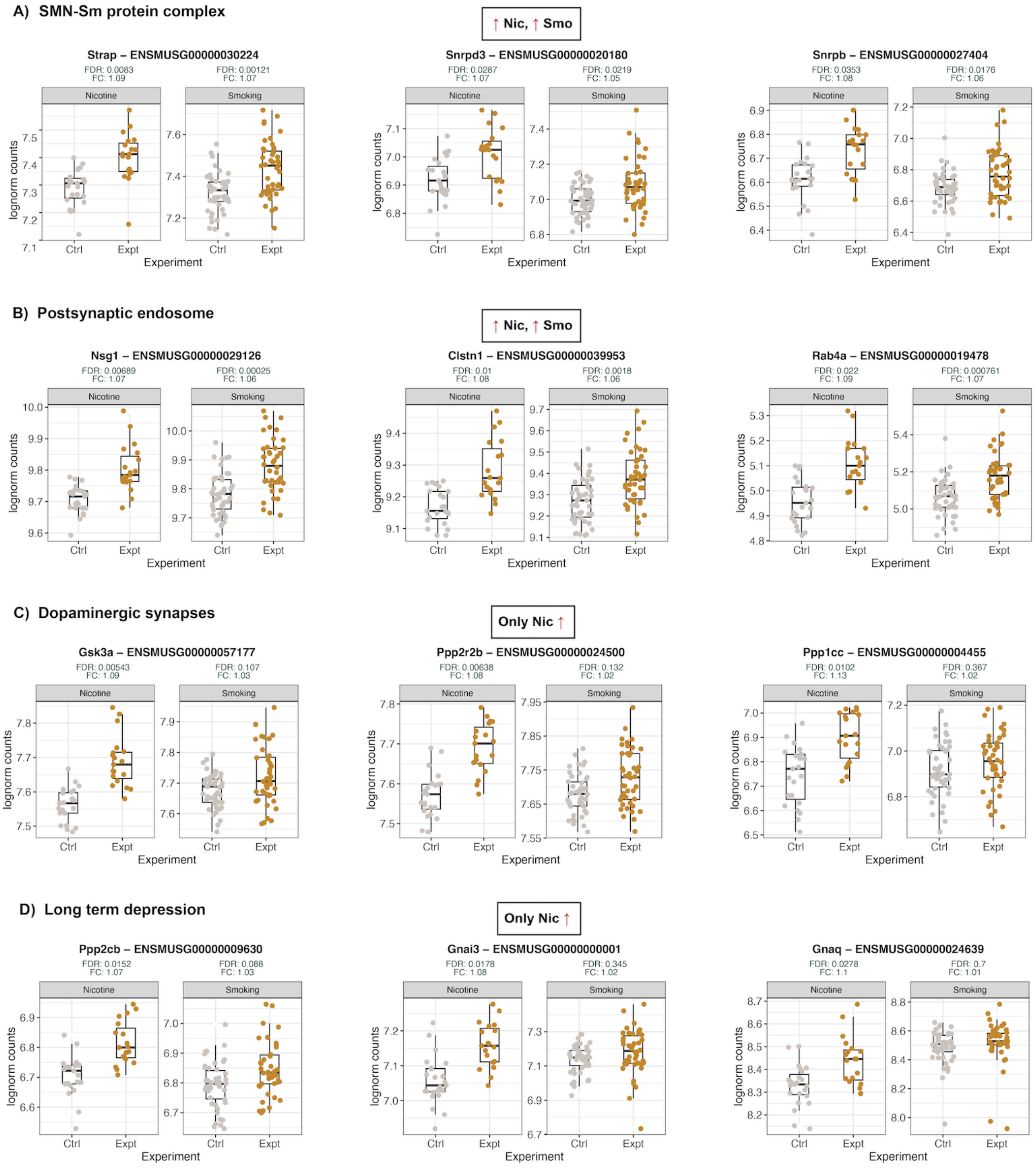

bioRxiv preprint doi: https://doi.org/10.1101/2024.11.05.622149; this version posted November 5, 2024. The copyright holder for this preprint (which was not certified by peer review) is the author/funder, who has granted bioRxiv a license to display the preprint in perpetuity. It is made available under aCC-BY 4.0 International license.

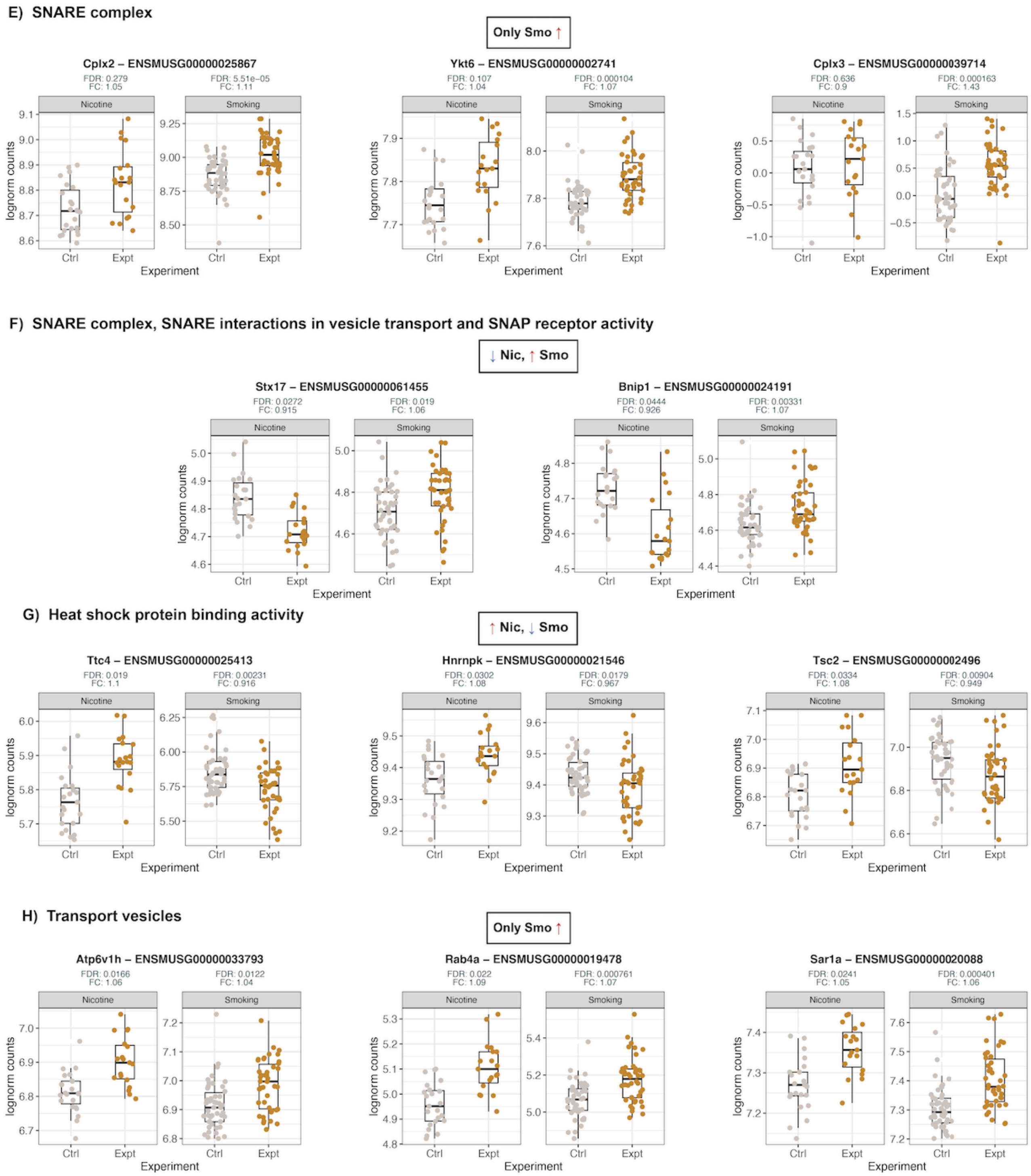

bioRxiv preprint doi: https://doi.org/10.1101/2024.11.05.622149; this version posted November 5, 2024. The copyright holder for this preprint (which was not certified by peer review) is the author/funder, who has granted bioRxiv a license to display the preprint in perpetuity. It is made available under aCC-BY 4.0 International license.

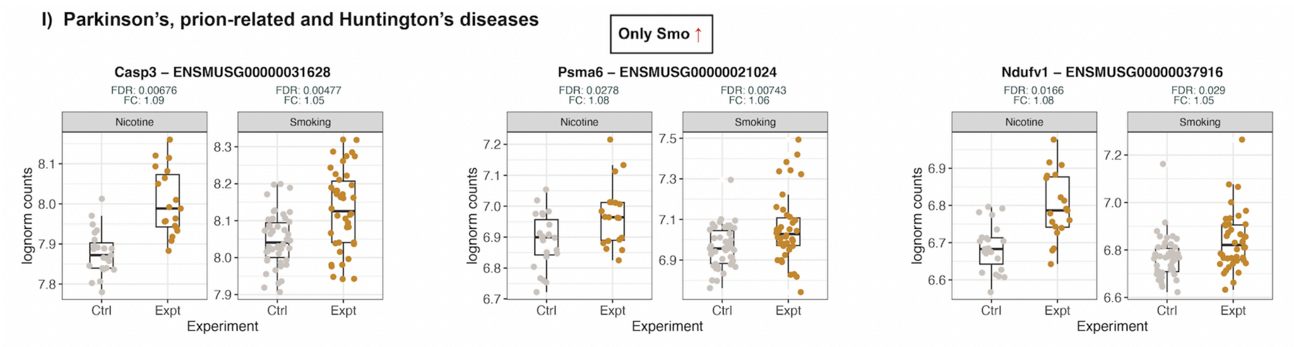

Supplementary Figure 15: **Expression of genes implicated in interest brain-related processes in pup brain**. Gene lognorm-counts of **A)** the top 3 most significant DEGs upregulated in nicotine and smoking-exposed samples that act in SMN-Sm protein complexes and **B)** postsynaptic endosomes. **C)** The top 3 most significant DEGs upregulated in nicotine samples only that are involved in dopaminergic synapses and **D)** in long-term depression. **E)** The top 3 most significant DEGs whose products work in the SNARE complex and are upregulated in smoking samples only (their DE transcripts too) or **F)** are up in smoking and down in the nicotine experiment and also have SNAP receptor activity and are implicated in SNARE interactions in vesicle transport. **G)** The top 3 DEGs upregulated for nicotine and downregulated for smoking whose gene products have heat shock protein binding activity. **H)** The top 3 most significant genes with DE transcripts that were upregulated only by cigarette smoke and act in transport vesicles and that are involved in **I)** Parkinson's, prion-related, and Huntington's diseases. Related to **Fig. 2** and **Fig. S14**.

FDR: false discovery rate; FC: fold-change; Ctrl: control samples; Expt: experimental (exposed) samples.

bioRxiv preprint doi: https://doi.org/10.1101/2024.11.05.622149; this version posted November 5, 2024. The copyright holder for this preprint (which was not certified by peer review) is the author/funder, who has granted bioRxiv a license to display the preprint in perpetuity. It is made available under aCC-BY 4.0 International license.

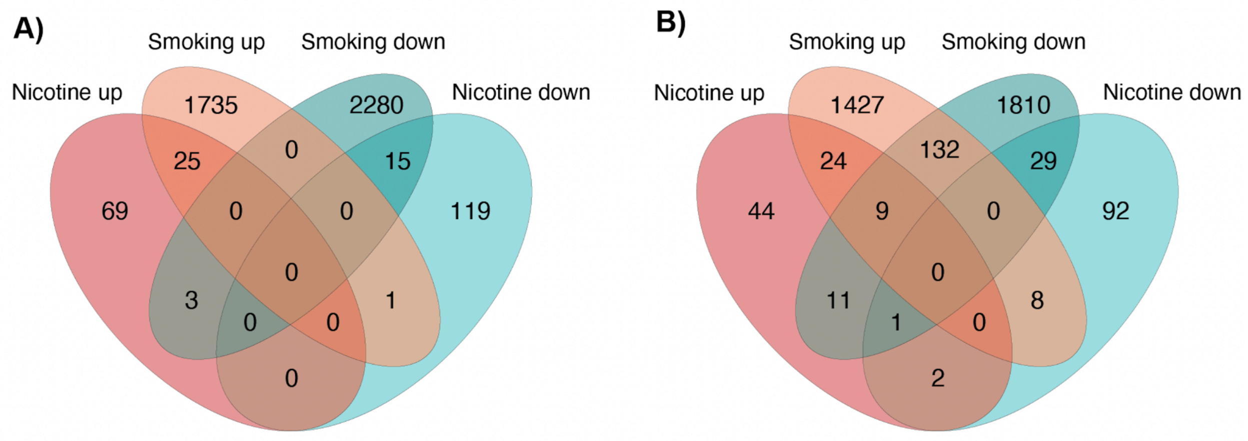

Supplementary Figure 16: Results of the DTE analysis in pup brain. Number of **A)** DE transcripts and **B)** genes with DE transcripts, up- and down-regulated in the nicotine and smoking experiments.

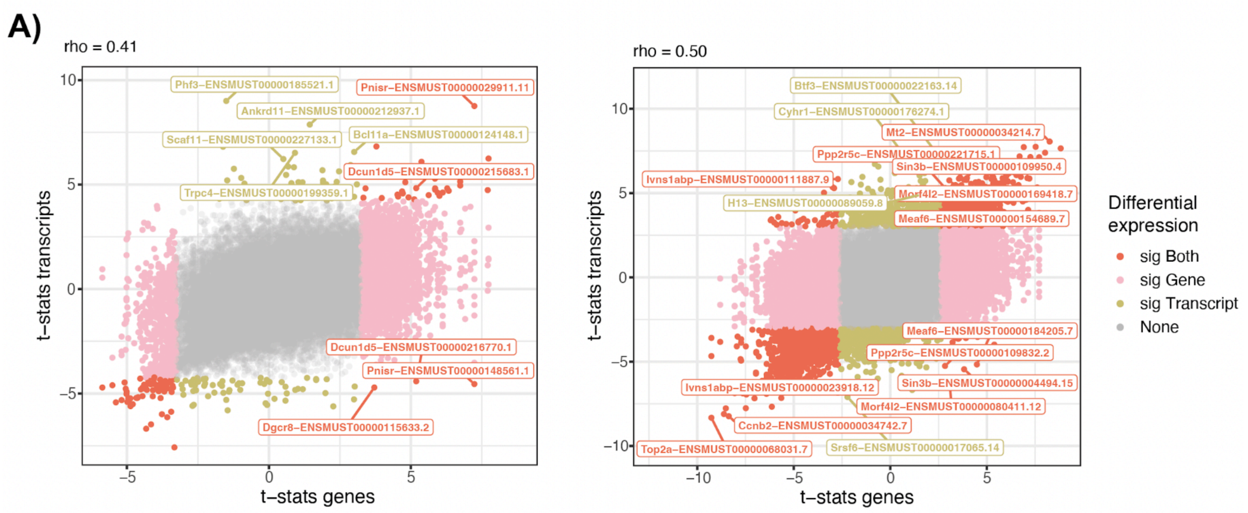

bioRxiv preprint doi: https://doi.org/10.1101/2024.11.05.622149; this version posted November 5, 2024. The copyright holder for this preprint (which was not certified by peer review) is the author/funder, who has granted bioRxiv a license to display the preprint in perpetuity. It is made available under aCC-BY 4.0 International license.

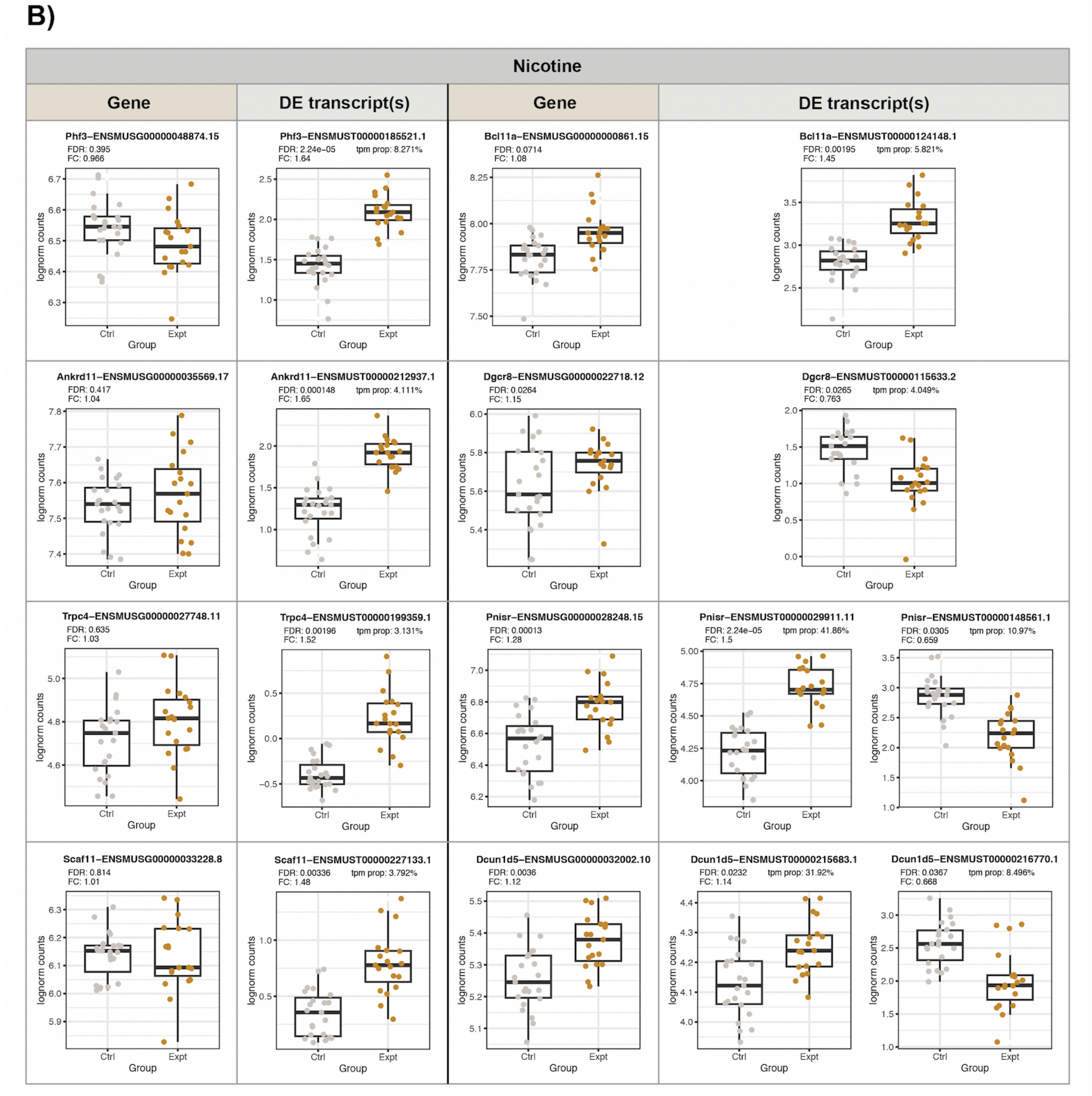

bioRxiv preprint doi: https://doi.org/10.1101/2024.11.05.622149; this version posted November 5, 2024. The copyright holder for this preprint (which was not certified by peer review) is the author/funder, who has granted bioRxiv a license to display the preprint in perpetuity. It is made available under aCC-BY 4.0 International license.

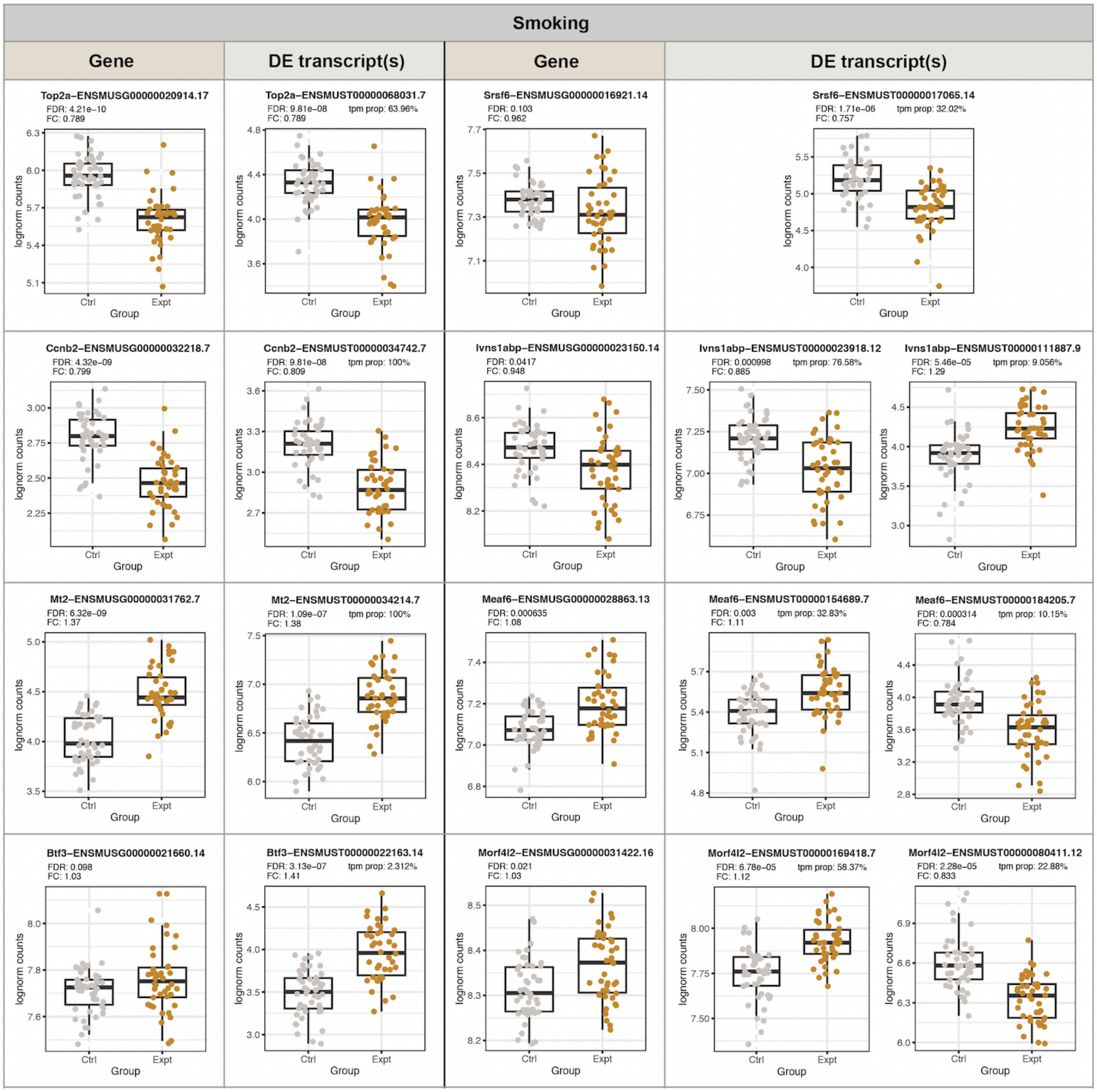

bioRxiv preprint doi: https://doi.org/10.1101/2024.11.05.622149; this version posted November 5, 2024. The copyright holder for this preprint (which was not certified by peer review) is the author/funder, who has granted bioRxiv a license to display the preprint in perpetuity. It is made available under aCC-BY 4.0 International license.

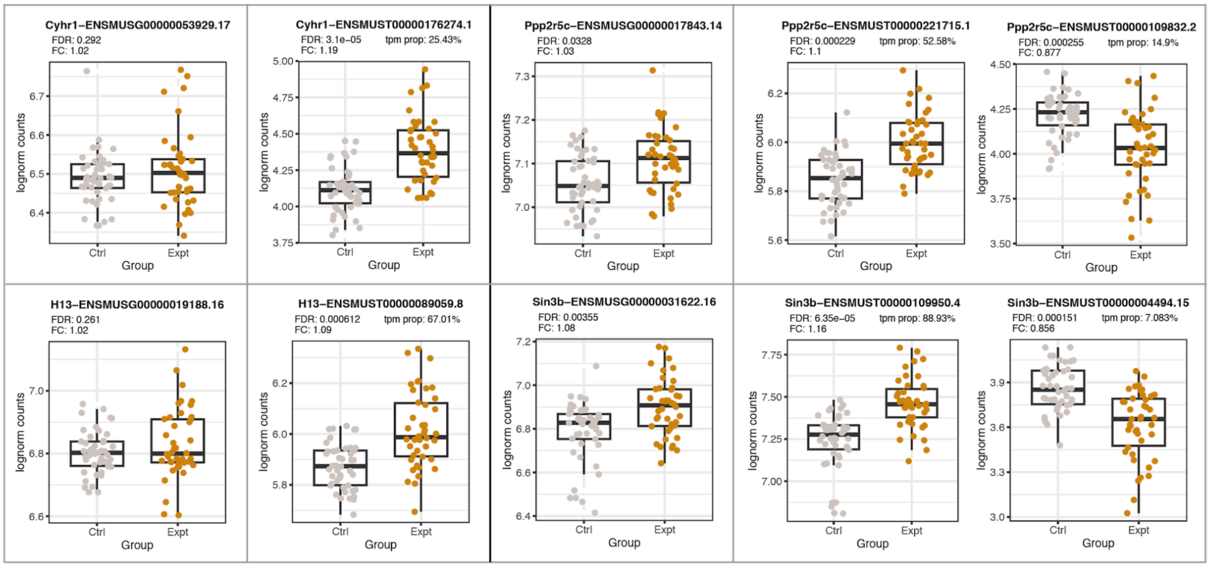

Supplementary Figure 17: **Expression of DE transcripts and their genes. A)** Plots showing the differential expression results as in **Fig. 3A** for prenatal nicotine vs vehicle exposure (left), and prenatal smoking exposure vs control (right). The significant DE transcripts of interest are labeled with their corresponding gene symbol and transcript Ensembl ID. **B)** Box plots show the expression of the labeled DE transcripts (in log-tpm) and their corresponding genes (in log-cpm) for the nicotine and smoking exposure. FDR: false discovery rate; FC: fold-change; tpm prop: the proportion of the total TPM of a gene that corresponds to the transcript. Total gene TPM was obtained adding TPM of all transcripts of the gene across all samples.

bioRxiv preprint doi: https://doi.org/10.1101/2024.11.05.622149; this version posted November 5, 2024. The copyright holder for this preprint (which was not certified by peer review) is the author/funder, who has granted bioRxiv a license to display the preprint in perpetuity. It is made available under aCC-BY 4.0 International license.

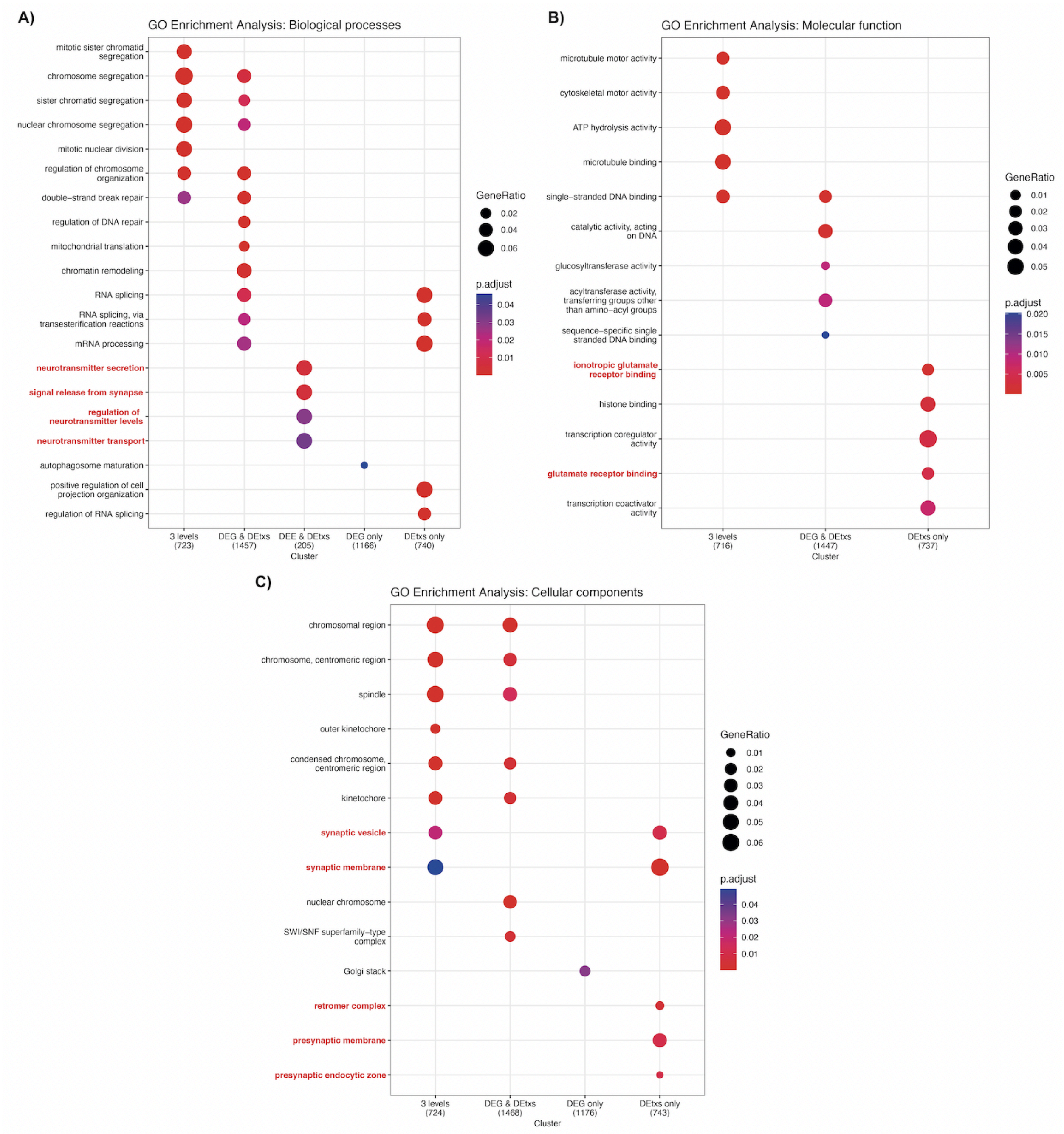

Supplementary Figure 18: **Functional enrichment analysis for genes with DE features in pup brain. A)** Biological processes, **B)** molecular functions, and **C)** cellular components significantly enriched (adjusted p-value<0.05) in clusters of DEGs with DE transcripts and DE exons (`3 levels`), DEGs with DE transcripts (`DEG & DEtxs`), DEGs with DE exons (`DEG & DEE`), non-DEGs with DE transcripts and DE exons (`DEE & DEtxs`), DEGs only (`DEG only`), and non-DEGs with DE transcripts only (`DEtxs only`) or DE exons only (`DEE only`) for the smoking experiment. The terms of interest appear in red. See **Fig. S14** caption for more details of these plots.

bioRxiv preprint doi: https://doi.org/10.1101/2024.11.05.622149; this version posted November 5, 2024. The copyright holder for this preprint (which was not certified by peer review) is the author/funder, who has granted bioRxiv a license to display the preprint in perpetuity. It is made available under aCC-BY 4.0 International license.

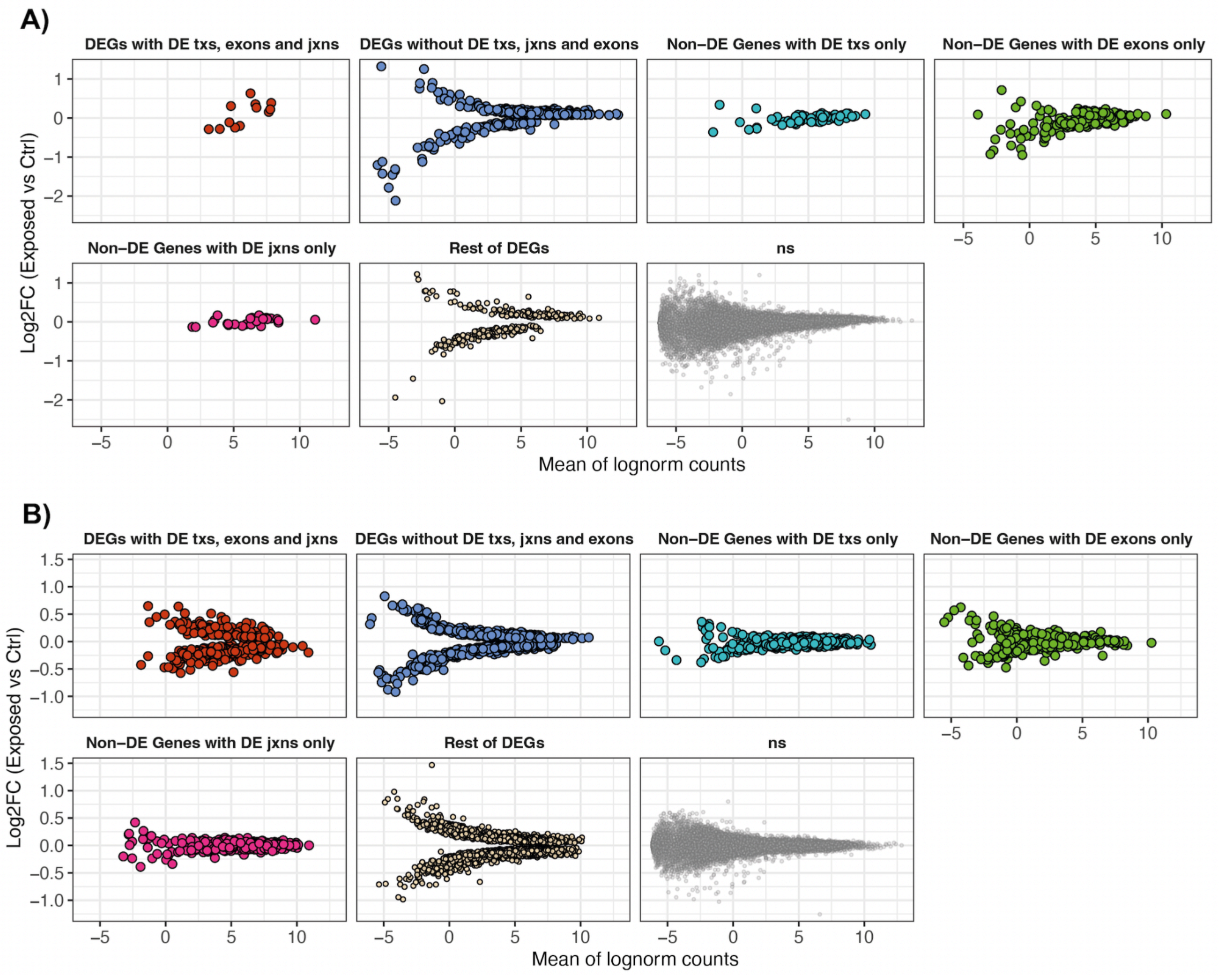

Supplementary Figure 19: MA plots of genes DE at different expression levels. Mean log-cpm and logFC of DEGs and non-DEGs with and without DE transcripts (txs), exons, and exon-exon junctions (jxns) for the **A)** nicotine and **B)** smoking exposure. Rest of DEGs are DEGs with two other DE features (txs and exons, txs and jxns, or exons and jxns).

bioRxiv preprint doi: https://doi.org/10.1101/2024.11.05.622149; this version posted November 5, 2024. The copyright holder for this preprint (which was not certified by peer review) is the author/funder, who has granted bioRxiv a license to display the preprint in perpetuity. It is made available under aCC-BY 4.0 International license.

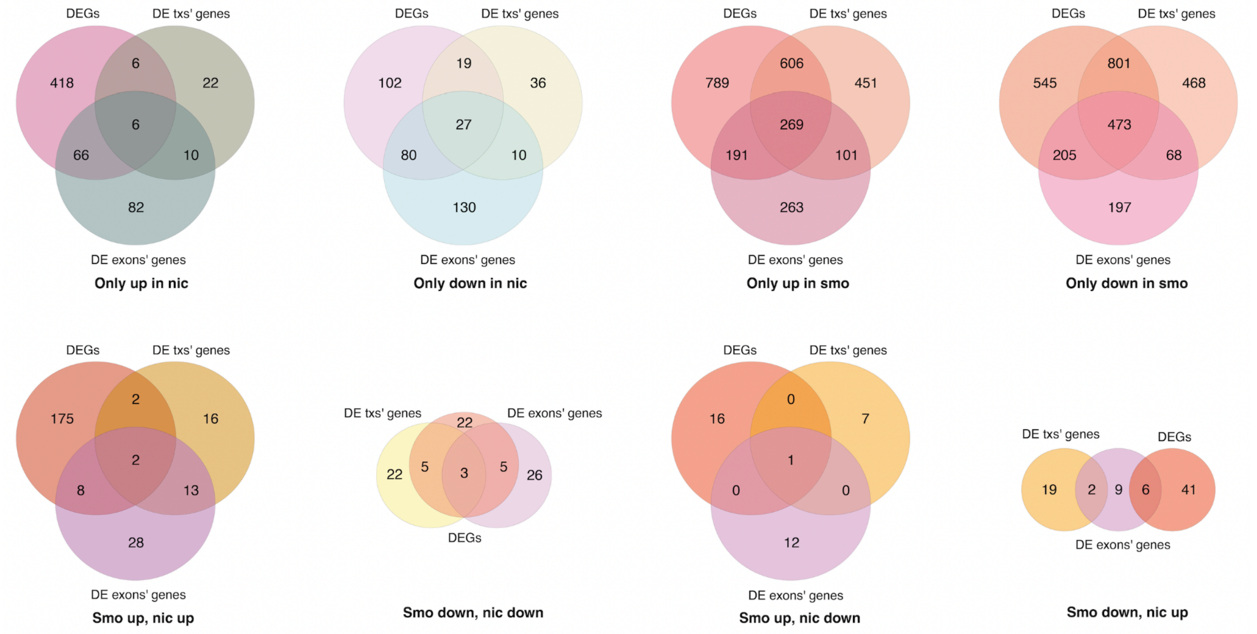

Supplementary Figure 20: **Comparison of DEA results at gene, transcript, and exon levels in pup brain.** Number of DEGs and genes of DE exons and DE transcripts (txs) are shown for the groups of up- and down-regulated features for the smoking and nicotine exposures as in **Fig. S14**.

bioRxiv preprint doi: https://doi.org/10.1101/2024.11.05.622149; this version posted November 5, 2024. The copyright holder for this preprint (which was not certified by peer review) is the author/funder, who has granted bioRxiv a license to display the preprint in perpetuity. It is made available under aCC-BY 4.0 International license.

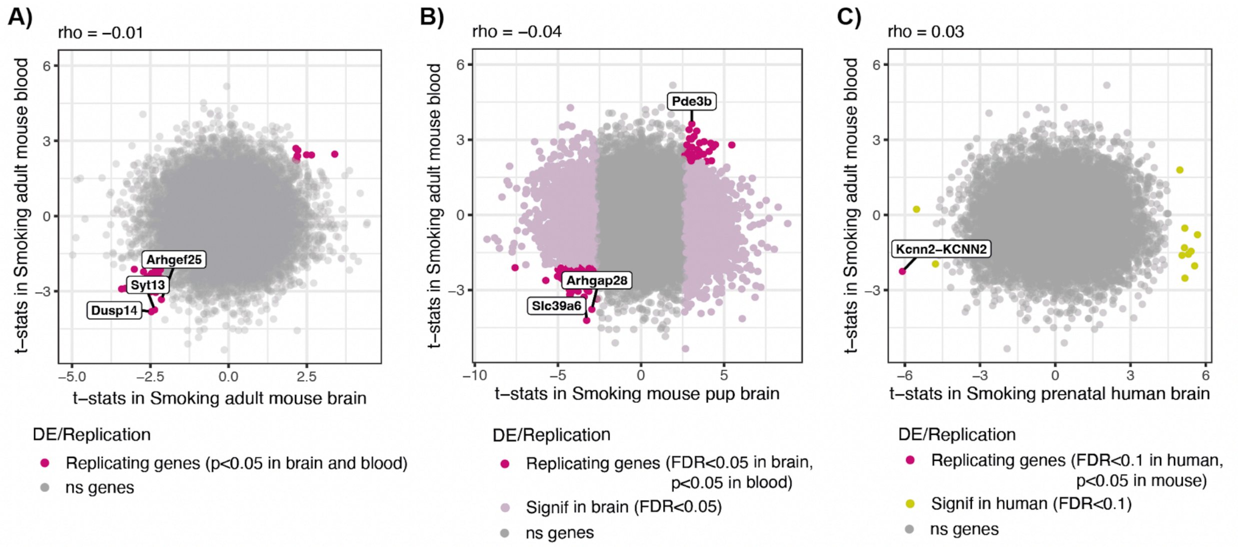

Supplementary Figure 21: Differential gene expression signal for smoking exposure in brain and blood. Comparison of the moderated *t*-statistics of the genes for DE by smoking exposure in adult blood vs **A)** adult brain, **B)** pup brain, and **C)** prenatal human brain. In dark pink genes that replicate in blood (with *p*-value<0.05 in adult brain/ FDR<0.05 in pup brain/ FDR<0.1 in human brain, *p*-value<0.05 in blood and with same logFC sign in both tissues). For B), in light pink DEGs in pup brain (FDR<0.05). For C), in yellow DEGs in human brain (FDR<0.1). In gray genes that were non-DEGs. The three replicating genes most significant in blood are labeled with their symbol in A) and B) and the unique replicating human gene in C) is labeled together with the symbol of the orthologous gene in mouse. Rho is the Spearman correlation coefficient between the *t*-stats. Related to **Table S5** and **Table S16**.

bioRxiv preprint doi: https://doi.org/10.1101/2024.11.05.622149; this version posted November 5, 2024. The copyright holder for this preprint (which was not certified by peer review) is the author/funder, who has granted bioRxiv a license to display the preprint in perpetuity. It is made available under aCC-BY 4.0 International license.

Supplementary File 1: additional DTE, DEE, and DJE results

Findings beyond the gene level for gestational nicotine and smoking exposure on developing frontal cortex

DTE results

In addition to the highly concordant DGE and DTE results for prenatal nicotine and smoking exposure (**Fig. 3A**), nicotine exposure caused changes in the expression levels of transcripts from the non-DEGs *Phf3, Ankrd11*, *Trpc4*, and *Bcl11a*, all expressed in the brain and with relevant roles in brain development (109–113), as well as the pyroptosis gene *Scaf11* that has been used in the prognosis of low-grade gliomas (114) and associated with Parkinson's disease (115) (**Fig. S17**, **Table S8**). On the other hand, highly consistent with DGE results, exposure to smoking led to the downregulation of *Top2a* and *Ccnb2*, and the upregulation of *Mt2* at the gene and transcript levels (**Fig. S17**, **Table S9**). Importantly, the human ortholog of *Ccnb2* promotes cerebral ischemic stroke and lung cancer by interacting with *TOP2A* (116). Furthermore, similar to nicotine exposure, for smoking exposure there were non-DEGs such as *Btf3*, *Cyhr1*, *H13* and *Srsf6* expressing DE transcripts (**Fig. S17**, **Table S9**). Interestingly, *Btf3* is essential for in utero embryonic development (117), whereas the splicing factor gene *Srsf6* has target genes involved in brain organogenesis and is likely to be responsible of missplicing events that lead to Huntington's disease (118,119); *Cyhr1* is known to be affected by chronic manganese (Mn) exposure that causes neurodegenerative changes in the frontal cortex (120), and the gene *H13* is crucial for embryonic development and brain morphology (121).

One interesting aspect was the identification of DE transcripts regulated in an opposite direction to that of their genes, as occurred with the nicotine-exposed DEG *Dgcr8* (**Fig. S17**, **Table S8**), as well as the presence DEGs with both up- and down-regulated DE transcripts within the same experiment, as was the case for *Pnsir* and *Dcun1d5* for nicotine, and *Meaf6*, *Ivns1abp*, *Morf4l2*, *Sin3b*, and *Ppp2r5c* for smoking exposure (**Fig. S17**, **Table S8**, **Table S9**). For these, transcripts going in the same direction as the gene accounted for a larger percentage of the total gene expression than transcripts with the opposite direction of regulation (**Fig. S17**), in line with past discoveries showing that genes tend to have dominant transcripts (122). Those genes could be subjected to differential transcript usage (DTU) in which not only their expression levels vary between conditions but also their splicing patterns change, resulting in different proportions of the expressed transcripts of a gene in one or the other condition. Future analyses of transcript expression proportions relative to the total expression of the genes will enable the inference of these events that can inform about substance exposure consequences at the transcriptional level that are disregarded by just analyzing DGE and DTE. Also, the actual posterior protein translation, post-translational modifications, and functional contribution of each individual DE transcript need to be explored.

bioRxiv preprint doi: https://doi.org/10.1101/2024.11.05.622149; this version posted November 5, 2024. The copyright holder for this preprint (which was not certified by peer review) is the author/funder, who has granted bioRxiv a license to display the preprint in perpetuity. It is made available under aCC-BY 4.0 International license.

DEE results

The functional enrichment analysis for genes with significantly regulated expression features further revealed non-DEGs that express DE transcripts and exons for smoking exposure that are implicated in neurotransmitter secretion and transport, regulation of neurotransmitter levels, and signal release from synapse (**Fig. S18A**), as well as products of DE transcripts of non-DEGs that present ionotropic glutamate and glutamate receptor binding activity and are part of the retromer complex, presynaptic membrane, and the presynaptic endocytic zone (**Fig. S18B,C**). Importantly, for smoking exposure there were DEGs with DE transcripts and exons whose protein products carry out their functions within synaptic vesicles and membranes (**Fig. S18C**). These additional neurological implications of gestational smoking exposure not identified by analyzing only DGE demonstrate how enriching it is to explore expression changes at these other expression feature levels (**Fig. S18**).

DJE results

DJE analysis was performed to find potentially novel splice isoforms, i.e., that are not annotated in GENCODE M25 (95,123). All DE exon-exon junctions except two for smoking exposure were novel, with at least one unannotated splice site or with an unknown combination of donor and acceptor sites. Of these, 5 and 201 DE junctions for nicotine and smoking exposure, respectively, were fully novel, with both splice sites unknown and without assigned gene. For these novel junctions their immediate following and preceding genes, as well as their nearest overlapping neighbor gene were located (see **Table S19**). The latter genes had a bigger overlap with the identified DEGs at the gene, transcript, and exon levels (**Fig. S22**), further supporting these nearest overlapping neighbor genes as bearers of potentially new isoforms, compared to the immediate upstream and downstream genes of DE junctions.

Together, DGE, DTE, DEE, and DJE results were concordant for both exposures in pup brain (**Fig. 3C**), with more highly expressed DEGs having DE transcripts, exons, and exon-exon junctions (**Fig. S19**), as well as DEGs with DE transcripts and DE exons, with all features regulated the same within each experiment (**Fig. S20**). Nonetheless, many DEGs only had significant DE signal at the gene level. One could hypothesize that these DEGs have low expression levels and thus, not enough reads to properly quantify their exons, transcripts, and junctions, but that was not necessarily the case: many of the DEGs with low mean expression didn't have other DE expression features (blue points in **Fig. S19** with mean lognorm counts < 0), but not all DEGs without significant features had low expression values; they had smaller logFCs (blue points in **Fig. S19**). On the other hand, there were also DE features from non-DE genes (**Fig. 3C**), where the high expression of such genes enabled the detection of some of their features as DE (**Fig. S19**). A plausible explanation for the presence of DE exons from non-DEGs or lowly-expressed genes is that during exon quantification reads mapping to regions shared by overlapping exons were assigned to all, inflating their number of reads and artificially increasing their expression (see **Supplementary Materials and Methods: Expression quantification**).

bioRxiv preprint doi: https://doi.org/10.1101/2024.11.05.622149; this version posted November 5, 2024. The copyright holder for this preprint (which was not certified by peer review) is the author/funder, who has granted bioRxiv a license to display the preprint in perpetuity. It is made available under aCC-BY 4.0 International license.

Notwithstanding, a series of caveats must be considered at the exon and exon-exon junction levels. One main limitation of analyzing exons is the lack of consistency between exon and transcript expression and the very different methods used to estimate their expression levels. Because shared exons are part of more than one transcript, their expression levels have a different impact when they are considered separately, as independent genomic features, instead of parts of multiple transcripts with different expression levels. In the case of overlapping exons, the current approach multi-counts the reads mapping to them, as previously mentioned (19). Thus, exon expression levels, when measured independently, do not necessarily correlate with the expression of the transcripts containing them, which is estimated using a different method that attempts to probabilistically resolve read mapping ambiguities for shared and overlapping exons across transcripts (98). As a result, expression counts of exons may be inaccurate and could lead to misleading inferences when projected to the transcript level. Similar considerations apply to junction level expression counts. For future analyses, transcript assembly could offer a better alternative to revealing previously uncharacterized isoforms.

bioRxiv preprint doi: https://doi.org/10.1101/2024.11.05.622149; this version posted November 5, 2024. The copyright holder for this preprint (which was not certified by peer review) is the author/funder, who has granted bioRxiv a license to display the preprint in perpetuity. It is made available under aCC-BY 4.0 International license.

Additional Supplementary Figures

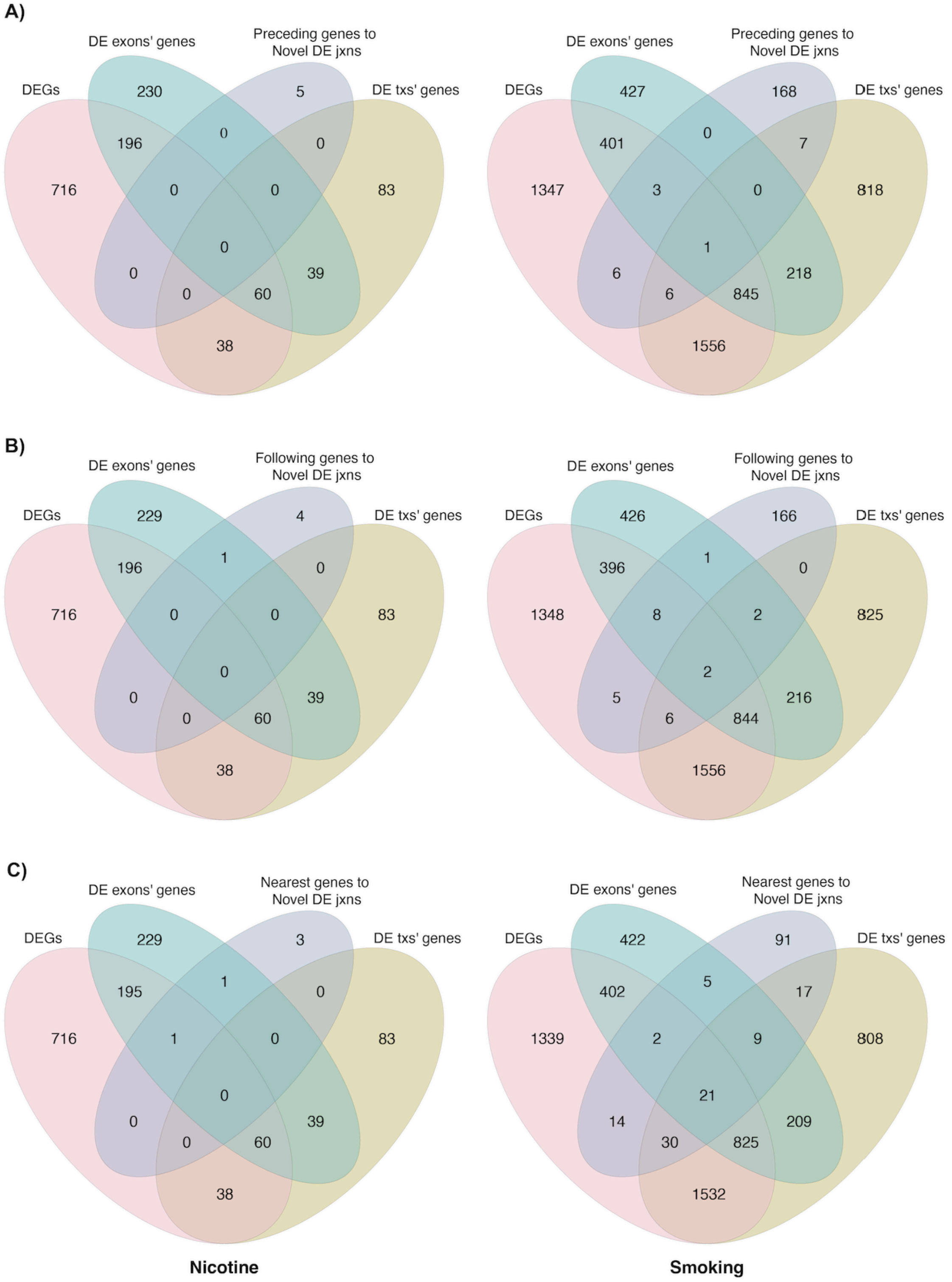

Supplementary Figure 22: Comparison of genes DE at different feature levels and genes associated with DE exon-exon junctions in pup brain. Overlap between DEGs, genes of DE exons,

bioRxiv preprint doi: https://doi.org/10.1101/2024.11.05.622149; this version posted November 5, 2024. The copyright holder for this preprint (which was not certified by peer review) is the author/funder, who has granted bioRxiv a license to display the preprint in perpetuity. It is made available under aCC-BY 4.0 International license.

genes of DE transcripts, and **A)** the preceding, **B)** following, and **C)** nearest genes to DE novel junctions without assigned gene, for nicotine (left) and smoking (right) exposure.

bioRxiv preprint doi: https://doi.org/10.1101/2024.11.05.622149; this version posted November 5, 2024. The copyright holder for this preprint (which was not certified by peer review) is the author/funder, who has granted bioRxiv a license to display the preprint in perpetuity. It is made available under aCC-BY 4.0 International license.

Supplementary Material References

95. Harrow J, Denoeud F, Frankish A, Reymond A, Chen C-K, Chrast J, et al. GENCODE: producing a reference annotation for ENCODE. Genome Biol. 2006 Aug 7;7 Suppl 1(Suppl 1):S4.1-9.

96. Frankish A, Diekhans M, Ferreira A-M, Johnson R, Jungreis I, Loveland J, et al. GENCODE reference annotation for the human and mouse genomes. Nucleic Acids Res. 2019 Jan 8;47(D1):D766–73.

97. Cotto KC, Feng Y-Y, Ramu A, Skidmore ZL, Kunisaki J, Conrad DF, et al. RegTools: Integrated analysis of genomic and transcriptomic data for discovery of splicing variants in cancer. BioRxiv. 2023 Jan 9;

98. Bray NL, Pimentel H, Melsted P, Pachter L. Near-optimal probabilistic RNA-seq quantification. Nat Biotechnol. 2016 May;34(5):525–7.

99. Robinson MD, McCarthy DJ, Smyth GK. edgeR: a Bioconductor package for differential expression analysis of digital gene expression data. Bioinformatics. 2010 Jan 1;26(1):139–40.

100. Robinson MD, Oshlack A. A scaling normalization method for differential expression analysis of RNA-seq data. Genome Biol. 2010 Mar 2;11(3):R25.

101. Collado-Torres L, Jaffe AE, Burke EE. jaffelab: Commonly used functions by the Jaffe lab. 2021;

102. McCarthy DJ, Campbell KR, Lun ATL, Wills QF. Scater: pre-processing, quality control, normalization and visualization of single-cell RNA-seq data in R. Bioinformatics. 2017 Apr 15;33(8):1179–86.

103. Hoffman GE, Schadt EE. variancePartition: interpreting drivers of variation in complex gene expression studies. BMC Bioinformatics. 2016 Nov 25;17(1):483.

104. Benjamini Y, Hochberg Y. Controlling the false discovery rate: a practical and powerful approach to multiple testing. Journal of the Royal Statistical Society: Series B (Methodological). 1995 Jan;57(1):289–300.

105. Lawrence M, Huber W, Pagès H, Aboyoun P, Carlson M, Gentleman R, et al. Software for computing and annotating genomic ranges. PLoS Comput Biol. 2013 Aug 8;9(8):e1003118.

106. Wickham H. ggplot2 - Elegant Graphics for Data Analysis. 2nd ed. Cham: Springer International Publishing; 2016.

107. Team RC. R: A Language and Environment for Statistical Computing. 2023;

108. Huber W, Carey VJ, Gentleman R, Anders S, Carlson M, Carvalho BS, et al. Orchestrating high-throughput genomic analysis with Bioconductor. Nat Methods. 2015 Feb;12(2):115–21.

109. Appel L-M, Franke V, Bruno M, Grishkovskaya I, Kasiliauskaite A, Kaufmann T, et al. PHF3 regulates neuronal gene expression through the Pol II CTD reader domain SPOC. Nat Commun. 2021 Oct 19;12(1):6078.

bioRxiv preprint doi: https://doi.org/10.1101/2024.11.05.622149; this version posted November 5, 2024. The copyright holder for this preprint (which was not certified by peer review) is the author/funder, who has granted bioRxiv a license to display the preprint in perpetuity. It is made available under aCC-BY 4.0 International license.

110. Ka M, Kim W-Y. ANKRD11 associated with intellectual disability and autism regulates dendrite differentiation via the BDNF/TrkB signaling pathway. Neurobiol Dis. 2018 Mar;111:138–52.

111. Fowler MA, Sidiropoulou K, Ozkan ED, Phillips CW, Cooper DC. Corticolimbic expression of TRPC4 and TRPC5 channels in the rodent brain. PLoS ONE. 2007 Jun 27;2(6):e573.

112. Gualdani R, Gailly P. How TRPC channels modulate hippocampal function. Int J Mol Sci. 2020 May 30;21(11).

113. Simon R, Wiegreffe C, Britsch S. Bcl11 transcription factors regulate cortical development and function. Front Mol Neurosci. 2020 Apr 8;13:51.

114. Shen L, Li Y, Li N, Zhao Y, Zhou Q, Shen L, et al. Integrative analysis reveals the functional implications and clinical relevance of pyroptosis in low-grade glioma. Sci Rep. 2022 Mar 16;12(1):4527.

115. Vázquez-Vélez GE, Zoghbi HY. Parkinson's disease genetics and pathophysiology. Annu Rev Neurosci. 2021 Jul 8;44:87–108.

116. Li M-J, Yan S-B, Chen G, Li G-S, Yang Y, Wei T, et al. Upregulation of CCNB2 and its perspective mechanisms in cerebral ischemic stroke and all subtypes of lung cancer: A comprehensive study. Front Integr Neurosci. 2022 Jul 19;16:854540.

117. Deng JM, Behringer RR. An insertional mutation in the BTF3 transcription factor gene leads to an early postimplantation lethality in mice. Transgenic Res. 1995 Jul;4(4):264–9.

118. Cabrera JR, Lucas JJ. MAP2 splicing is altered in huntington's disease. Brain Pathol. 2017 Mar;27(2):181–9.

119. Sathasivam K, Neueder A, Gipson TA, Landles C, Benjamin AC, Bondulich MK, et al. Aberrant splicing of HTT generates the pathogenic exon 1 protein in Huntington disease. Proc Natl Acad Sci USA. 2013 Feb 5;110(6):2366–70.

120. Guilarte TR, Burton NC, Verina T, Prabhu VV, Becker KG, Syversen T, et al. Increased APLP1 expression and neurodegeneration in the frontal cortex of manganese-exposed non-human primates. J Neurochem. 2008 Jun;105(5):1948–59.

121. Wilson R, Geyer SH, Reissig L, Rose J, Szumska D, Hardman E, et al. Highly variable penetrance of abnormal phenotypes in embryonic lethal knockout mice. [version 2; peer review: 3 approved]. Wellcome Open Res. 2016;1:1.

122. Gonzàlez-Porta M, Frankish A, Rung J, Harrow J, Brazma A. Transcriptome analysis of human tissues and cell lines reveals one dominant transcript per gene. Genome Biol. 2013 Jul 1;14(7):R70.

123. Frankish A, Diekhans M, Jungreis I, Lagarde J, Loveland JE, Mudge JM, et al. GENCODE 2021. Nucleic Acids Res. 2021 Jan 8;49(D1):D916–23.
